# Challenges and Opportunities of MicroRNAs in Lymphomas

**DOI:** 10.3390/molecules190914723

**Published:** 2014-09-17

**Authors:** Giacoma De Tullio, Vincenza De Fazio, Nicola Sgherza, Carla Minoia, Simona Serratì, Francesca Merchionne, Giacomo Loseto, Angela Iacobazzi, Antonello Rana, Patrizia Petrillo, Nicola Silvestris, Pasquale Iacopino, Attilio Guarini

**Affiliations:** 1 Haematology Unit, National Cancer Research Centre, Istituto Tumori “Giovanni Paolo II”, Bari 70124, Italy; 2 Haematology and Bone Marrow Transplantation Unit, Antonio Perrino Hospital, Brindisi 72100, Italy; 3 Medical Oncology Unit, National Cancer Research Centre, Istituto Tumori “Giovanni Paolo II”, Bari 70124, Italy; 4 Clinical Institute “Prof. R. De Blasi”, Reggio Calabria 89100, Italy

**Keywords:** miRNAs, target therapy, lymphoid development, Hodgkin lymphoma, non-Hodgkin lymphoma, prognostic biomarker, therapeutic targets, antagomirs

## Abstract

MicroRNAs (miRNAs) are small non-coding RNAs that control the expression of many target messenger RNAs (mRNAs) involved in normal cell functions (differentiation, proliferation and apoptosis). Consequently their aberrant expression and/or functions are related to pathogenesis of many human diseases including cancers. Haematopoiesis is a highly regulated process controlled by a complex network of molecular mechanisms that simultaneously regulate commitment, differentiation, proliferation, and apoptosis of hematopoietic stem cells (HSC). Alterations on this network could affect the normal haematopoiesis, leading to the development of haematological malignancies such as lymphomas. The incidence of lymphomas is rising and a significant proportion of patients are refractory to standard therapies. Accurate diagnosis, prognosis and therapy still require additional markers to be used for diagnostic and prognostic purpose and evaluation of clinical outcome. The dysregulated expression or function of miRNAs in various types of lymphomas has been associated with lymphoma pathogenesis. Indeed, many recent findings suggest that almost all lymphomas seem to have a distinct and specific miRNA profile and some miRNAs are related to therapy resistance or have a distinct kinetics during therapy. MiRNAs are easily detectable in fresh or paraffin-embedded diagnostic tissue and serum where they are highly stable and quantifiable within the diagnostic laboratory at each consultation. Accordingly they could be specific biomarkers for lymphoma diagnosis, as well as useful for evaluating prognosis or disease response to the therapy, especially for evaluation of early relapse detection and for greatly assisting clinical decisions making. Here we summarize the current knowledge on the role of miRNAs in normal and aberrant lymphopoiesis in order to highlight their clinical value as specific diagnosis and prognosis markers of lymphoid malignancies or for prediction of therapy response. Finally, we discuss their controversial therapeutic role and future applications in therapy by modulating miRNA.

## 1. Introduction

The term “lymphoma” encompasses at least 48 distinct types of malignancy that vary in clinical behavior, morphologic appearance, and immunologic and molecular phenotype. Insights into the understanding of the molecular and cellular mechanisms involved in lymphoma formation and progression, as well as the role of the microenvironment and immune system, have enhanced the accuracy of patient risk-stratification and identified new potential therapeutic targets. Patients who require treatment often receive some types of chemotherapy, radiotherapy, immunotherapy, or a combination of these. In most cases, this approach is effective at the beginning, but it is often complicated by significant short- and long-term side effects. Unfortunately, aggressive lymphomas relapse, indolent slow-growing lymphoma transformations are not uncommon, and salvage therapy is often associated with progressive resistance. Therefore, despite improved outcomes, for example through the incorporation of monoclonal antibodies or with intensive chemotherapy, malignant lymphomas remain incurable in several histotypes and are one of the leading causes of cancer-related death. The pathologic classification of lymphoproliferative disorders continues to evolve, reflecting new insights into the cells of origin and the biological bases of these heterogeneous diseases. Therefore, based on the WHO classification, treatment is determined by identifying the specific lymphoma type and, if relevant, by considering tumor grade and other prognostic factors. However, in spite of progress in the accuracy of lymphoma diagnosis, the criteria for distinguishing different lymphoma entities sometimes overlap as some lymphoma types exhibit significant clinical and molecular heterogeneity. As a result of this, it may be difficult to distinguish between different lymphoma entities and therefore to personalize therapeutic strategies. Accordingly, the integration of more sensitive and reliable biomarkers for appropriate clinical use is a critical need.

With respect to biomarker detection, the characterization of a recently discovered class of regulatory small molecules, such as microRNAs (miRNAs), is an emerging field of study. MiRNAs are small, non-coding RNA molecules that control the expression of many target messenger RNAs (mRNAs) involved in normal cell functions (differentiation, proliferation and apoptosis). Their aberrant expression and/or function are related to the pathogenesis of many human diseases such as cancers. In particular, a growing body of evidence indicates that mRNAs are contributing factors in many cellular processes including lymphoid differentiation, lymphoma pathogenesis and progression. Indeed, not only their aberrant expression or function has been associated with lymphoma pathogenesis, but also, as expected, almost all lymphoid malignancies seem to have a distinct and specific miRNA profile, thereby highlighting the diagnostic role of any distinct and specific miRNA signature. The profiling of miRNA expression shows probably its best, and certainly the most immediate, potential use as novel sensitive biomarker for lymphoma diagnosis and prognosis. Recently, highly stable free circulating miRNAs have been discovered in peripheral whole blood, plasma and serum, and can be used as circulating markers. Since much evidence suggests that some miRNAs are involved in drug resistance or sensibility, analysis of circulating miRNAs in particular has great potential for detecting early relapse and identifying patients at risk for poor response, as well as for predicting and monitoring treatment response. Considering that the immune system is an essential player for the disease progression and pathogenesis of some lymphomas, another reason for the increased interest in miRNAs is their use as promising tools for the assessment of immune cell activation and the evaluation of various inflammatory responses. Ultimately, perhaps the most exciting imminent opportunities offered by miRNAs are their potential as novel therapeutic molecules, as either antagonists or agonists to specific cell types, in order to inhibit lymphoma cell survival and/or drug resistance mechanisms.

## 2. MicroRNA Biogenesis and Mechanism of MicroRNA Gene Regulation

**Figure 1:**
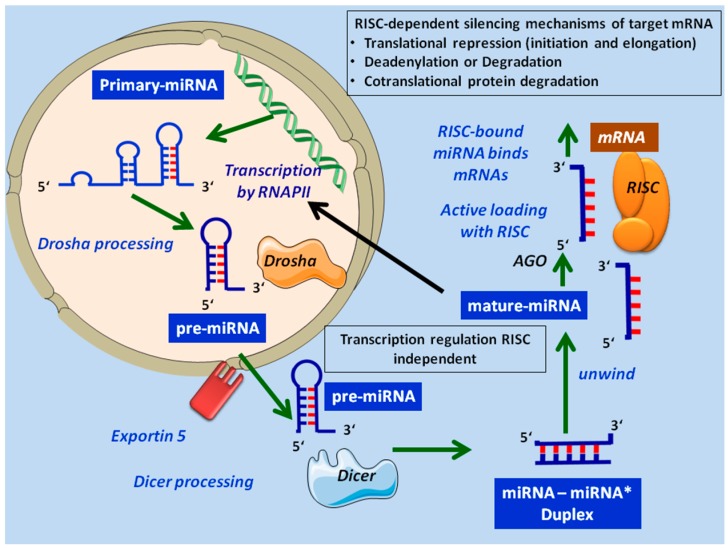
Canonical biogenesis pathways of microRNAs. RISC, RNA Interfering silencing complex.

miRNAs are small, evolutionary conserved, non-coding RNA molecules of around 22 nucleotides (nt) in length, which can regulate gene expression at the post-transcriptional level by translational silencing or by impairing the stability of their target mRNAs. MiRNAs are initially transcribed as a long precursor in the nucleus by RNA polymerase II (although there are instances of polymerase III transcription) [1,2] from unique miRNA genes, which can be located in intergenic regions as well as in the exons or introns of other genes named “host” genes. Most miRNAs are generated via a canonical mechanism (Figure 1), although there are various non-canonical pathways that bypass the microprocessor step. The initial products of transcription—sometimes of several hundred or thousand nt, capped at the 5′ end and polyadenyladed at the 3′ end—are called primary-miRNAs (pri-miRNAs) and are able to function both as pri-miRNAs and mRNAs [3]. These precursors, structurally similar to miRNAs, enter into a microprocessor complex that contains RNase III endonuclease Drosha [4,5,6] and are processed into a hairpin precursor (approximately 70–100 nt) known as pre-miRNAs. They are subsequently exported to the cytoplasm by exportin-5 (Exp5) [7,8]. Once in the cytoplasm, the double pre-miRNA strand is cleaved by Dicer, another RNase III endonuclease, to form a short miRNA: miRNA* duplex which is then unwound by helicase into a single mature miRNA strand (21- to 22-nt) and passenger miRNA.

Interestingly, multiple miRNAs can be produced within a single pri-miRNA transcript, each of which can act independently. This mechanism is greatly elucidated by miR-17-92 cluster, whose individual miRNA components undergo a selective biogenesis under given biological context [9]. The specific biogenesis of individual miRNAs within a polycistronic miRNA cluster increases the complexity levels in the regulation of miRNA expression and function in different cell types and under different contexts. Accordingly, this polycistronic structure of miRNA cluster genes elucidates the unique capacity and specificity of miRNAs to regulate the complex molecular networks involved in the development and disease in cell- and context-dependent manner. Furthermore, it is plausible that post-transcriptional silencing mediated by each miRNA component within the polycistronic structure may also be regulated in a cell type- and context- dependent manner.

Mature miRNA is directly embedded with Argonaute protein to form RNA Interfering Silencing Complex (RISC). In animals, the RISC-bound miRNA binds mRNAs through a sequence that at least partially matches the 3′-untraslated regions (3′-UTR) of target mRNA. The regulation mechanism of the translation depends on the degree of miRNA-mRNA complementarity: direct cleavage and degradation when the complementarity is perfect (in plants); protein translation blocking/inhibition in the case of imperfect base pairing (in mammals).

It is estimated that miRNAs regulate up to 60% of all human genes [10] by targeting multiple transcripts. Most importantly, miRNAs virtually regulate all cellular processes including cell cycle, developmental timing, cell proliferation, apoptosis, cell differentiation, metabolism, organ development and haematopoiesis [11,12]. In this context, a great effort has been made to clarify their role in normal and malignant haematopoiesis. In particular, studies on the whole genome miRNA (miRome) have demonstrated that it changes dynamically during lymphoid maturation, as it is tissue- and stage-specific as well as temporally regulated [12].

Because of their important role in the differentiation and proliferation of different hematopoietic lineages, it is not surprising that miRNAs have been implicated in immune cell functions as well as the secretion of cytokines in microenvironments. Indeed, recent studies have documented that miRNAs have unique expression profiles in the cells of both innate and adaptive immune systems, and play significant roles in the regulation of cell development and function [13,14,15,16,17].

## 3. MicroRNAs as Regulators of Lymphoid Maturation

Lymphoid differentiation is a complex process in which lymphoid progenitors originating from the hematopoietic stem cell (HSC) differentiate themselves into increasingly specialized cell populations through asynchronous expression of gene products, enabling them to respond to environmental cues resulting from interactions with the immune system. This process is highly regulated by a combination of transcription factors, epigenetic modifications, miRNAs, and extrinsic signals. In particular, miRNAs appear critical in almost every stage of lymphoid differentiation, with some stage-specific miRNAs that modulate the ability of the lymphoid cells to respond to environmental signals [18,19].

The key roles of miRNAs in lymphoid cell biology and their involvement in lymphopoiesis have been partly elucidated by the results of two main experimental approaches:

1. Evaluation of global expression patterns of miRNAs in specific cell lineages, and comparison of these profiles in several stages of differentiation and/or in normal cells *vs.* lymphoma cells.
2. *In vitro and in vivo* functional and mechanistic studies of miRNAs carried out by (a) replacing or knockdown of miRNAs or (b) silencing only specific single miRNA-mRNA target interactions through a mutation in complementary sites to the 3′-UTR or (c) using chemically-modified antisense oligonucleotides, termed antimiRs, which hold the mature miRNA in competition withtarget mRNAs leading to functional inhibition of the miRNA and repression of the direct targets.

Functional studies are the most useful approach to identify the miRNAs potentially relevant for both the development and function of lymphoid cells, and consequently to determine their role in lymphoma formation and progression. However, identifying the direct involvement of a given miRNA in a specific pathway is not always easy, because each miRNA regulates many mRNA targets and the same mRNA can be regulated by one or more miRNAs. Consequently, the possible indirect effects mediated by other miRNAs can be difficult to rule out. Nevertheless, functional studies have confirmed the importance of miRNAs in lymphomagenesis and have identified which among them were the potential actors specifically implicated in each phase of lymphoma development.

First of all, the clearest evidence of the global importance of miRNA regulatory mechanisms has been obtained by blocking the biogenesis of mature miRNAs. Several investigators have demonstrated that these small molecules have a crucial role in lymphocyte homeostasis, since if they are absent the development and the differentiation of lymphocytes cannot proceed. Furthermore, their findings have helped to know that not all steps of lymphopoiesis are equally and strictly dependent on the presence of miRNAs and that their role is different in each developmental stage and lineage.

The most popular functional approach used to identify physiologically important miRNAs *in vivo* are animal models in which concomitant loss of multiple miRNAs can be produced by deletion of Dicer in the germline (straight knock-out) or in defined tissues (conditional knock-out). Over a hundred studies have investigated the straight and conditional knockout mice of Dicer [20], and collectively they have shown different implications during the sequential stages of development. The effect of Dicer deletion in mice germline is a lethal phenotype with a premature death at embryonic day 7.5 and loss of detectable multipotent stem cells, suggesting that the absence of miRNAs is incompatible with life [21]. Moreover, conditional Dicer deletion in murine embryonic stem cells makes these cells unable to differentiate [22], suggesting that miRNAs are required in hematopoiesis. Further functional studies in individual lymphocyte cell lineages have highlighted that both the Dicer-dependent miRNA pathway and several miRNAs are critical drivers for lymphoid precursor cell fate decisions and for regulation of their functions. These studies also showed that miRNA expression patterns change throughout normal lymphopoiesis from multipotent progenitors (MPP) to common lymphoid progenitors (CLP) as well as from pro- to pre-lymphocyte in primary lymphoid organs, and during the subsequent TCR and BCR repertoire evolution.

Although not reviewed in this article, miRNAs have also shown the capacity to modulate, directly or indirectly, the expression of multiple lineage-specific genes during the activation of innate immune cells (macrophages, dendritic, and natural killer cells). In the following sections, we review the role of miRNAs during the development and differentiation of adaptive immune cells, emphasizing information from individual miRNAs and miRNA clusters that are involved in the malignant transformation process and that could be markers or targets for therapeutic gene silencing in the more common types of lymphoma.

## 4. Role of miRNAs in B-Cell Maturation

Lymphoid cell production occurs through the differentiation from primitive pluripotent hematopoietic stem cells (HSCs) within the bone marrow (BM) by highly regulated multiple developmental stages. The HSCs give rise to uncommitted hematopoietic precursors, known as multipotent hematopoietic progenitors (MPPs). According to the popular model (classical dichotomy model), MPPs undergo a dichotomous lineage restriction into common myeloid progenitors (CMPs) and common lymphoid progenitors (CLPs), able to give rise to all the lymphoid lineages [23]. From these lineages, mature B or T-cells originate through a network of transcriptional regulators. The recent identification in both mice and humans of lymphoid progenitors that possess myeloid, lymphoid and NK cell lineage potential (LMPPs) implies however that the potential to generate myeloid cells is also retained in the lymphoid branches (myeloid-based model).

**Figure 2:**
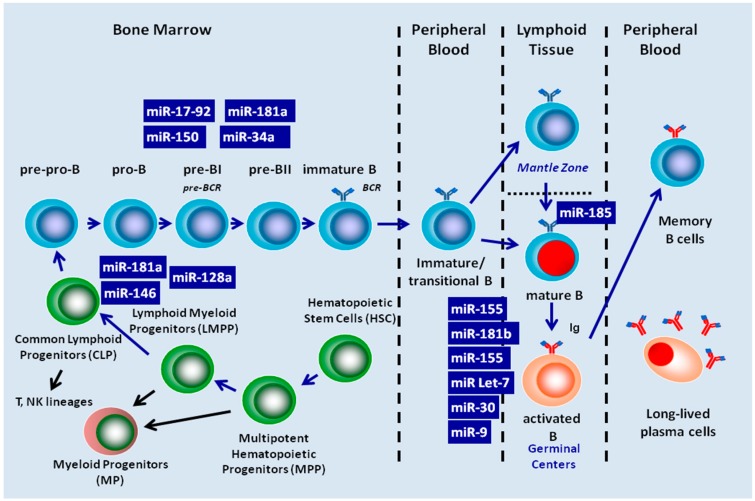
MiRNAs Involved in the regulation of B-cell development from common lymphoid progenitor cell to produce memory and plasma cell.

As regards B-cell development, the CLP compartment is likely a major source of downstream B-cells and contains cells already committed to the B-cell lineage. Expression in mice of the B-cell marker B220 by a subset of CLPs (known as pre-pro-B cells or CLP-2s) coincides with their entry into the B-cell-differentiation pathway. Subsequently, pre-pro-B progenitor cells progress through well-defined stages, each characterized by distinct biological features: pro-B, precursor B (pre-BI, Large pre-BII, and small pre-BII), immature and transitional or naïve B cells that migrate from the BM to lymphoid organs where they are transformed, in the context of T cell-dependent germinal centers (GC), into mature B lymphocytes, and finally into memory and/or antibody-secreting plasma cells (Figure 2) [24,25,26,27]. The functional role of each miRNA in the different stages of B-cell differentiation has mostly been elucidated through experiments carried out on genetically modified mice (Table 1).

### 4.1. miRNA Control of B-Cell Development in Bone Marrow

Although gene expression during lymphocyte development is driven primarily by transcription factors, an additional level of regulation is mediated by miRNAs. An approach to globally address the importance of miRNA control in B-cell development has been used to assess the effects of Dicer knockouts in B cell progenitors [28]. Depending on the development stage (early or later B-cell stage) in which it occurs, Dicer deletion has different consequences. Conditional deletion of Dicer in early B-cell progenitors induces an almost complete block of the pro- to pre-B cell transition with a greatly reduced number of B cells in the BM and peripheral lymphoid organs.

**Table 1:** The main miRNAs involved in the different stages of B-cell maturation and lymphomas listed with their functions and regulatory mechanisms.

miRNAs	Direct/Indirect Target	Function/Ref.	Regulation	Lymphomas/miRNAs involved/Ref.
miR-17-92	• Bim • *E2F1* • *PTEN* • Akt • mTOR • *PHLPP2*	Control of transition from proB- to preB-cells. Enhancement of cell survival and inhibition of apoptosis by targeting *PTEN* and Bim, antagonizing with *BCL2* [29,30]. Positive regulation by *MYC* and simultaneous repression of *E2F1* expression by miR-17-5p and miR-20a: generation of *MYC*/miR-17-92/*E2F1* circuit that accelerates the development and increases the aggressiveness of the tumor [31,32,33,34,35]. miR-19b represses apoptosis and promotes cell proliferation and angiogenesis by repressing *PTEN* expression and function, thus resulting a functional activation of Akt/mTOR pathway via PI3K pathway [36]. Induction of chemoresistance in MCL by activating the *PI3K/AKT* pathway trough targeting of *PHLPP2* [37]. *VEGF* up-regulate the expression levels of only miR-18, miR-19 and miR-20 to participate in the control of angiogenic phenotypes [9]. Selective miR-17-92 biogenesis and likely post-transcriptional silencing mediated by each miRNA component within miR-17-92 may be regulated in a cell type- and context- dependent manner [9,38].	• c*-MYC*: positive regulation at transcriptional level [33] • *E2Fs*: positive regulation by direct binding [34,39] • *TP53*: repression under hypoxia conditions and at post-transcriptional level [9] • *VEGF*: up-regulates only miR-18, miR-19 and miR-20 at post transcriptional level [9]	• DLBCL (miR-17-5p, miR-19b): mainly in GC subtype [31,40,41] • MCL (miR-17-3p, miR-17-5p, miR-18a) [27,31,34,35,37,42] • B-CLL/SLL (miR-19a, miR-19b, miR-92) [43] • BL (miR-17-miR-3p, miR-18a, miR-19a, miR-19b, miR-92) [27,43,44] • HL (miR-17-5p, miR-92 [43] • FL [41]
miR-181a	• *BCL2*	Involvement in commitment to Lymphoid cell fate, B and T-cell differentiation, and specifically promotion of early B-cell development [19,45]. Block of human progenitor cell differentiation [46].		• MCL [47] • B-CLL [48]
miR-181b	• *AID*	Modulation of somatic hyper-mutation and class-switch recombination together with miR-155 but at different stage of B-cell activation [48,49].		B-CLL [48]
miR-150	• *c-MYB* • *DKC1* • *AKT2*	Control of transition from proB- to preB-cells [50,51]. Down-regulation of *c-MYB*, expression [18,51]: interaction important for oncogenesis and (or) tumor progression [52,53,54]. Regulation of the NK and iNKT cell development [55]. Reduction of phosphorylated *AKT* (ser473/4) levels and increasing Bim *and TP53* by directly down-regulating the *DKC1* and *AKT2* expression [56].		• MCL [57] • B-CLL [18,58] • BL [18,58] • DLBCL [41,59]
miR-185	• *BTK*	BCR development.		
miR-155	• *AID* • *PU.1* • *HGAL* • *SMAD5* • *INPP5D (SHIP1)*	Control of B-cell differentiation and GC reaction: activation and function of B cell in germinal centres and for T-cell dependent antibody responses [19,46,60,61,62] by negatively modulating somatic hypermutation and class-switch recombination through the targeting of *AID* [62] and *PU.1* [63]. Down-regulation of *HGAL* expression, leading to a decreasing of RhoA activation and increasing of spontaneous and chemoattractant-induced lymphoma cell motility [64] Oncomir that regulates proliferation and enhances cell survival by: • escaping TGF-β growth-inhibitory effects trough destroying *SMAD5* activity [64,65] • promoting TNFalpha-dependent growth of B cell trough target of *INPP5D (SHIP1)* [66,67,68,69,70] when overexpressed (DLBCL)	• c-*MYC*: negative regulation at post transcriptional level.	• DLBCL [58,64,65,66,70,71,72,73] • HL [71,72] • B-CLL [71] • MCL [57] • BL [58,74,75,76] • FL [41]
miR-34a	• *FOXP1* • *SIRT1 (TP53)* • *c-MYC*	Growth suppressive function and pro-apoptotic effect in pro-B cells of which it controls the transition to pre-B stage by *targeting FOXP1* [77,78] know as B cell on cogene. [79] It is negatively regulated *by c-MYC* [80] and positively regulated by *TP53* [80,81,82,83,84]. In turn, by inhibiting SIRT1, it actives *TP53* resulting apoptotic effects mediated by TP53*/SIRT1*/miR-34a pathway. Epigenetically silenced in many lymphomas, mainly NK/T-NHL [55].	• *TP53*: positive regulation at transcriptional level [84] • *c-MYC*: epigenetically silenced	• Malignant transformation of MALT to gastric DLBCL [78] • NK/T-NHL [55]
miR-9	• *PRDM1* • E cadherin	Regulation of B-cell terminal differentiation into plasma cells and memory B cells [85,86] It regulates E-cadherin [58]	• *MYC/MYCN*: positive regulation through direct binding to the miR-9-3 locus	• BL [58] • B-CLL/SLL [58] • HL [60] • FL [41,55] • DLBCL [55,58]
miR-30	• *BCL6* • *PRDM1*	Regulation of B-cell differentiation by determining the ability of developing B cells to move to the GC [50]		• DBLCL [85]
Let-7	• *PRDM1* • *RAS*	Regulation of B-cell terminal differentiation into plasma cells and memory B cells [85,86]		• HL [86] • FL [87] • DBLCL [88]
miR-29	• *TCL-1* • *MCL-1*	Down-regulates *TCL1* and *MCL1* expression [48,89]		• B-CLL [48,90,91] • (ALK+)-ALCL [89]

B-CLL, B Chronic lymphocytic leukemia; DLBCL, Diffuse large B-cell lymphoma; HL, Hodgkin lymphoma; MCL, Mantle cell lymphoma; MZL, Marginal zone lymphoma; BL, Burkitt's lymphoma; FL, Follicular lymphoma; NK/T Ly, Natural Killer/T lymphoma; ALCL, Anaplastic large-cell lymphomas.

This block in B-cell development is caused by a strong induction of the apoptosis of pro-B cells partially mediated by a significant up-regulation of Bim (known also as BCL2L11, *Bcl-2-like protein 11*), a pro-apoptotic protein which antagonizes with anti-apoptotic molecules such as *B-cell lymphoma 2*, *Bcl-2* [12,19]. *Bim* is target of different miR-17-92 cluster members (also known as Oncomir-1). This cluster contains several individual miRNAs, frequently amplified and overexpressed in lymphomas and various solid tumors. In particular, after the deletion of Dicer, miR-17-92 is undetectable in both pro- and pre B-cells [12]. Accordingly, the effects of Dicer deletion mainly depend on the loss of miR-17-92 cluster.

Several miRNA families control B-cell development. Numerous studies have documented that specific expression profiles of several miRNAs can be linked to the different steps of the maturation/differentiation process. The most important step of regulation is the transition from pro-B to pre-B-cells, which is regulated mainly by four miRNAs: miR-181, miR-150, miR-17-92 cluster, and miR-34a. They control a relatively limited number of key targets mostly involved in cell death, cell survival, and cell proliferation. The miR-181 family is composed of miR-181-a-1/miR-181-b-1, miR-181-a-2/miR-181-b-2, and miR-181-c/miR-181-d clusters, which can be regarded as positive regulators during early haematopoiesis and lineage commitment of both B and T cells [92]. It is thought that miR-181 acts independently in the T and B-cell lineages, performing different functions in each such as repressing different target genes. With regard to B-cell differentiation and examining the expression levels of this miRNA family in hematopoietic organs [19], a highly dynamic kinetic has been observed. Its expression was very low in undifferentiated progenitor cells, increased to high levels in early B-cells, but dramatically decreased from the pro-B to pre-B-cells, finally resulting very low in lymph nodes. This expression profile and more specifically the lower expression in undifferentiated lymphocytes is in agreement with the statement that miR-181 along with miR-128-a and miR-146 expression is a general feature of commitment to a lymphoid cell fate [46]. Chen *et al.* [19] studied the effects of the ectopic expression of miR-181 *in vitro* and *in vivo* in murine HSCs. They showed that it promoted the increase of the fraction of B cell lineage (CD19) cells in parallel with a substantial decrease of T-lymphoid cells, particularly the CD8 T cell subset, with a relatively small or insignificant decrease of CD4+ T cells and without affecting myeloid lineage cells. These findings were concurrent with the preferential expression of miR-181 in B-lymphoid cells of mouse BM. They suggest a fine modulation in timing of miR-181, which acts as a very important, positive, regulator for the differentiation of B cells, as demonstrated by the drastic reduction of its expression already in the pre B stage [19].

The molecular mechanism and functions of miR-150 have been well studied in lymphopoiesis models and in a variety of cancers, including B-cell lymphomas and B-cell chronic lymphocytic leukemia (B-CLL). This miRNA is expressed in all lymphoid tissues, including lymph nodes, the spleen and the thymus, where it shows a dynamic expression profile during lymphocyte development, as it is highly expressed in mature B and T-cells but not in their progenitors or upon their activation [50]. In particular, when ectopically expressed in transgenic mouse B-cell progenitors, the miR-150 significantly impaired the pro-B to pre-B-cell stage transition, partially by increasing apoptosis of the pro-B-cells, with a consequent significant reduction of mature B-cells in peripheral blood and lymphoid tissues and a moderate enhancement of T lymphopoiesis and myelopoiesis [51]. Conversely, deletion of miR-150 in mouse germline resulting consequent complete absence in all cells generated homozygous mutant mice (*miR-150^−/−^ mice)* that were viable and fertile, with a normal development of B and T-cells but showing an expansion of one of the mature B-cell subset (CD19+B220loCD5+CD43+CD23- the so called B1a cells) in the spleen and in the peritoneal cavity along with the enhancement of both humoral and T-cell dependent immune responses [18]. This expansion could be due to the miR-150 loss-of-function in early B-cell development stages, or in B1-cells, or both. In any way, this was in striking contrast to mice with a B-cell-specific deletion of *c-MYB* in which B1-cells were absent. By using loss- and gain-of-function gene targeting approaches for miR-150 it is possible to study the functional inter- play between miR-150 and *c-MYB in vivo.* In particular, if *c-MYB* was *in vivo* targeted by the miR-150, as predicted, the deficiency of this latter will lead to a phenotype opposite to that caused by the deletion of *c-MYB* and the ectopic expression of miR-150 in B-cell progenitors will result in block of B-cell development. Indeed, it was previously demonstrated that B-cell specific deletion of *c-MYB* gene led to a severe blocks of B–cell development at the pro- to pre-B transition and disappearance of mature B1-cells [93]. Given the dependence of the B1 subset on *c-MYB* [93] and indeed its precise dosage in the different cell subsets (purified peripheral lymph node CD4 T-cells and B2-cells, and sorted peritoneal cavity B1a-cells) determined by western blot [18], it has been suggested, therefore, that the expansion of B1-cells in miR-150-deficient mice mainly depend on down-regulation of *c-MYB* expression in a dose-dependent manner at some stage of development involving activation of these cells and this dramatically affects lymphocyte development and response. *c-MYB* is a proto-oncogene protein belonging to the *MYB* (*myeloblastosis)* family of transcription factors, which directs multiple steps of early lymphoid development and is implicated in human leukaemogenesis and other cancers. During lymphocyte commitment and maturation, *c-MYB* expression surprisingly reflects that of miR-150, since it is highly expressed in progenitors and down-regulated in mature naive lymphocytes in mice [18]. In line with this, opposite patterns of miR-150 and *c-MYB* expressions have been observed in immortalised cell lines and in B-cell subsets of human tonsil tissues [52,94]. Finally, the experimental induction of the ectopic expression of miR-150 in breast cancer, leukemia cells, and a Burkitt’s lymphoma cell line led to reduced endogenous *c-MYB* gene transcription at both the mRNA and protein levels. This confirms that *c-MYB* is an evolutionary conserved target of miR-150 and implies that miR-150/*c-MYB* interaction may also be important for oncogenesis and (or) tumor progression [52,53].

The transition from pro-B to pre B-cells is also regulated by miR-17-92 cluster comprising seven miRNAs located at chromosome 13q31-32, normally highly expressed in B and T progenitor cells and down-regulated after maturation [12]. The function of miR-17-92 in normal B-cell development was investigated in order to clarify its role in the pathogenesis of B-cell lymphomas. This cluster is essential for B-cell development as it specifically promotes the pro-B to pre-B transition (pre B Cell Receptor checkpoint), enhancing the survival of the B-cell at this stage by dampening the expression of the pro-apoptotic protein Bim. As demonstrated by Ventura *et al.* [29] in mice the absence of miR-17-92 led to increased levels of the Bim and inhibited B cell development at the pro-B to pre-B transition in hematopoietic cells. Indeed, flow cytometry analysis of fetal liver cells from miR-17-92 deficient mice embryos demonstrated a greatly reduced percentage and absolute number of circulating B-cells. This significant and specific reduction was due to an enhanced cell death of specifically miR-17~92-deficient pro-B-cells as demonstrated by cell cycle analysis. The authors demonstrated that members of the miR-17~92 cluster regulate survival of early B cell progenitors by repressing the expression of the pro-apoptotic *BIM* gene at the pro- to pre-B-cell transition. In miR-17-92 deficient pro- and pre-B-cells from fetal livers they observed a significant increase of Bim protein levels compared to control and associated with the presence of the two binding sites in the 3′UTR of Bim. Furthermore, Bim play a role in controlling B lymphocyte apoptosis and in suppressing *Myc*-induced B cell lymphomagenesis. Finally as Bim, know also as BCL2L11, antagonizes with *BCL2* they demonstrated the ability of *BCL2* overexpression, induced by *BCL2* transgene, to partially rescue B-cell development defect due to miR-17-92 deficiency and consequent up-regulated Bim expression. These findings suggested a possible mechanism through which deletion or overexpression of miR-17~92 affects B-cell development and lymphomagenesis. In contrast, in another study, mice that overexpressed miR-17-92 in their lymphocytes exhibited severe lymphoproliferative disorders and autoimmunity [30], due to both the enhancement of cell proliferation and the inhibition of the apoptotic pathway of *Activation-Induced Cell Death* (*AICD*). The reduced apoptosis is due to the decreased Bim expression along with down-regulation of *Phosphatase and Tensin homolog* (*PTEN*), another miR-17-92 target, which acts as a tumor suppressor gene during lymphomagenesis. Concomitant with the impairment of B development, there was an increase of peripheral CD4+ T cells together with high production of IFN and IL-10 by activated CD4+ T cells and elevated secretion of serum of IgG2a and IgG3 [30]. Interestingly, the miR-17-92 cluster is positively regulated by c*-MYC* with which it acts to accelerate tumor development [31]. Indeed, the presence of a *MYC*/miR-17-92/*E2F1* circuit has also been demonstrated. In this circuit c*-MYC* up-regulates the miR-17-92 cluster and simultaneously activates *E2 Factor 1* (*E2F1*), a transcription factor promoting cell-cycle progression. The expression of *E2F1* is in turn negatively regulated by two individual components of the cluster, miR-17-5p and miR-20-a [32,33]. In addition to c-*MYC*, the transcription of miR-17-92 is directly activated by *E2F1* and *E2F3*. The last one is the major *E2F* family member that binds to the mir-17-92 promoter region [34,39]. *E2Fs* are essential for the progression of the cell cycle, activating a large number of S phase genes. These finding are consistent with the functions of mir-17-92 in promoting proliferation in a variety of cell types such as lymphoid. Consequently, the cycling cells are likely to have elevated miR-17-92 due to the periodic burst of *E2F* activity during S phase, while quiescent cells may have reduced miR-17-92 levels. These findings clearly show the mechanism trough which c-*MYC* tightly controls the proliferative signal by activating *E2F1* transcription and simultaneously by inactivating *E2F1* translation through a miRNA-based mechanism [33]. More recently, miR-19 has been identified as one of the main oncogenic components of the miR-17-92 cluster [36]; it directly represses *PTEN* and, consequently activates the *Ak strain of Thymona /Mammalian target of rapamycin m*TOR (Akt/mTOR) pathway, thus leading to the enhancement of cell survival during lymphomagenesis [33]. Indeed the Akt /mTOR pathway may be overactive because *PTEN* is faulty or deficient. Overall, the miR-17-92 cluster participates in a number of pathways, including Bim, *PTEN*, Akt/mTOR, *c-MYC* and *E2F1*. This explains the frequent involvement of this cluster in the pathogenesis of several lymphomas.

Interestingly, Yan *et al.* [95] showed miR-17-92 as novel target for *Tumor Protein p53* (also known as *TP53*) mediated gene repression, most apparent in cell under hypoxia. As miR-17-92 exert a strong effect to promote cell survival, it is likely that repression of miR-1792 by *TP53* give rise to apoptosis under hypoxia treatment [95]. More studies elucidate that this cluster is not only regulated by a network of transcriptional machineries, but it is likely also subjected to intricate regulation through a largely unknown mechanism which modulate the expression of individual miRNAs inside the cluster. For example, Suarez *et al.* [9] demonstrated that *Vascular Endothelial Growth Factor* (*VEGF)* mediated up-regulation of miR-17-92 only increasing the expression of three individual miRNA components of the cluster, miR-17, miR-18 and miR-20, to participate in the control of angiogenic phenotypes. This suggests a selective miR-17-92 biogenesis under given biological contexts [9]. Previously, Guil-Caceres *et al.* [38] observed that miR-18 biogenesis specifically required the RNA binding protein hnRNPA1, indicating a potential connection between signal transduction and specific miRNA biogenesis. The specific biogenesis of individual miRNAs within the miR-17-92 cluster results the complex regulation undergo by this cluster and elucidates its capacity to regulate different process in cell-dependent manner. It is also plausible that post-transcriptional silencing mediated by each miRNA component within miR-17-92 may also be regulated in a cell type- and context- dependent manner.

In addition to miR-17-92 and miR-150, miR-34-a also regulates the pre B cell receptor checkpoint. Contrarily to miR-17-92 however, it exhibits pro-apoptotic effects and, like miR-150, has growth-suppressive functions in pro-B-cells in which it is highly expressed [12]. In particular constitutive expression of miR-34a in HSCs leads to a phenotype characterised by a reduction in the number of mature B-cells as a result of the blockage in pro-B to pre-B transition that stops pre-B generation [77]. Employing both loss and gain-of-function of miR-34a in transgenic mice has shown that the phenotype associated with the over-expression of miR-34a depends on the repression of *Forkhead box protein P1 (FOXP1)* [77], a transcription factor, known as B cell oncogene, required for early B-cell development [78] and considered a highly specific target of miR-34a during B-cell differentiation [77,78]. Conversely, loss of miR-34a function resulted in increased numbers of mature B-cells accompanied by modestly elevated amounts of *FOXP1*. As confirmation, recently Carig *et al.* [96] revealed that the malignant transformation of *Mucosa associated lymphoid tissue* (MALT) lymphoma to gastric *Diffuse Large B-Cell Lymphoma* (DLBCL) is linked to overexpression of *FOXP1* due to the repression of the tumor suppressor miR-34a mediated by the aberrant expression of *MYC* in high-grade transformation of gastric B-cell lymphoma. It is thought that the effects of miR-34a on *FOXP1* may be the mode through which *TP53* suppresses this potentially oncogenic protein in post-germinal center B-cells. Indeed miR-34a is also intimately connected to *TP53* through a feedback loop in which *TP53* induces miR-34 expression that in turn activates p53 through *Sirtuina 1* (*SIRT1*) inhibition. This suggests that increasing the amount of miR-34a could enhance therapeutic apoptosis due to the tumor suppressive role of this miRNA [81]. This is consistent with the observation that miR-34a is localised in a locus (1p36) frequently lost in many solid tumor types and haematological malignancies, including lymphomas.

More recently Okada *et al.* [82] demonstrated that miR-34a represses *HDM4*, a potent negative regulator of *TP53*, creating positive feedback loop acting on *TP53.* MiR-34a deficiency alone however does not exhibit a strong oncogenic effect, as confirmed in Kras-induced mouse lung cancer model. Conversely though, it strongly promotes tumor-genesis when *TP53* is haploinsufficient. These findings reveal that miR-34 deletion alone is insufficient to induce tumorgenesis possibly due to the considerable redundancy in the *TP53* pathway, and it is the defectiveness of the *TP53*-miR-34 feedback loop that can enhance oncogenesis in a specific context. The intricate cross-talk between *TP53* and miR-34a highlights an important tumor suppressor effect generated by this positive feedback loop. On the other hand, it has also been shown that c-*MYC* down-regulates miR-34a expression. In turn, by a negative feedback loop, miR-34a can down-regulate c*-MYC*, as well as *TP53*, via the *Cyclin-Dependent Kinase Inhibitor 2A and Murine Double Minute 2 CDKN2A-MDM2* pathway [47,83] so that in B lymphoid cells that concomitantly over express c*-MYC* the expression of *TP53* is down-regulated in a *MYC*-dependent manner [79]. This finding suggests another pathway through which c-*MYC* exerts its pathogenic mechanism in the onset of lymphoma. Together these findings suggest the importance of miR-34a in tumor pathogenesis and appear to offer a broad range of therapeutic opportunities. Therefore, it is likely that a miR-34-based therapy may be among the first miRNA mimics to reach the clinic [12,79].

### 4.2. miRNA Regulate Mature B-Cell Activation and Functions

Following the successful rearrangement of light chain genes, the pre B cells differentiate into immature B-cells when whole IgM molecules are expressed as functional B Cell Receptors (BCR) on the cell surface. These newly formed immature B cells, if self-tolerant, leave the bone marrow and begin to recirculate between the blood, the secondary lymphoid tissues and the lymph as transitional/immature stage. Of note, differently from mice, B-cells that are produced in human BM are fully functional and their complete maturation does not depend on the spleen. In lymph nodes where the immature/transitional B-cells migrate (the other secondary lymphoid organs have a similar microanatomy and function), they are positively selected by contact with their specific antigen and become activated, and through the secretion of chemokines by the distinct stromal cell network they are recruited in B cell follicles and in the surrounding T cell zones. Within the follicles, B cells proliferate rapidly forming the highly specialized microenvironment of germinal centers (GC). Inside the GC, proliferating B cells undergo an affinity maturation process that is the result of the somatic hypermutation and class-switch recombination of Ig-genes, followed by selection of high affinity B-cells by an antigen displayed on follicular dendritic cells (FDCs). High affinity B-cells emerging in GC give rise to long-lived plasma cells and memory B-cells. Among the various mechanisms regulating B-cell maturation, somatic hypermutation is a crucial checkpoint as the antibody repertoire depends on it. Therefore, it is not surprising that this is the main stage regulated by miRNAs (Figure 2). Like in bone marrow B-cell development, miRNAs both as whole and as specific single miRNA are recognized as important players in peripheral and antigen-dependent B-cell development. For instance, Dicer deletion in mature B-cells leads to the expansion of the mantle zone B-cell compartment (MZ) along with the impaired generation of follicular B-cells (FO) and a further complete disruption of the antibody repertoire, characterised by accumulation of self-reactive antibodies and autoimmunity [12]. Gain-of-function and miRNA profiling approaches have also revealed that an impaired phenotype characterized by a bias generation of B cell subsets results in the loss of miR-185 and in up-regulation of its target, *Bruton tyrosine kinase* (*BTK*), which is a key component of *BCR* signaling [97].

A miRNA that play a pivotal role in this stage is miR-155, even though its importance is not directly linked to lymphoid differentiation. Its central role is mainly related to functional involvement in several aspects of the adaptive immune system where it modulates T and B *in vivo* responses [60,61]. MiR-155 is processed from a non-coding RNA known as the *B-cell integration cluster* (*BIC*), which is encoded within the exon of a gene originally isolated near a common retroviral integration site in avian leucosis virus-induced lymphomas. Its expression is induced upon stimulation of the antigen receptor on B and T lymphocytes, as well as stimulation of the *Toll-like receptor* (TLR) of macrophages and dendritic cells (DCs) [98]. Accordingly miR-155 is widely expressed in different immune cells including B and T cells, DCs and macrophages, thereby supporting its crucial role in immune system functions. This has been confirmed by the observation that miR-155 deficient mice exhibit defective responses of both innate and adaptive arms of immunity, especially with regard to the less humoral responsiveness to immunization. These defects are concomitant with the decreased numbers of B-cells in the germinal centre and the reduction of both immunoglobulin M (IgM) and class-switched antigen-specific antibodies [60,63]. It has been hypothesized that this immunodeficiency mainly depends on the impaired ability of DCs lacking miR-155 to activate T-cells, which is due to their defective antigen presentation capacity and abnormal co-stimulatory functions [60]. Moreover, using both animal models, Thai *et al.* [61] found that miR-155 was necessary for the activation and function of B-cells in germinal centres, and for T-cell dependent antibody responses. This is consistent with high miR-155 expression in B- and T-activated cells in germinal centres [61,62]. MiR-155 negatively modulates somatic hypermutation and class-switch recombination of the immunoglobulin locus by specifically modifying a binding site in 3′-UTR of the *activation-induced cytidine deaminase (AID*), a master regulator required for Ig gene diversification in B-lymphocytes and for *MYC*-related translocations [49]. Since the generation of a diversified antibody repertoire depends on the correct regulation of Ig gene diversification, the interaction between miR-155 and *AID* is a key to determining the normal functions of B-cells. Interestingly, it has been shown that when this interaction in mice is destroyed by introducing mutations in the putative miR-155 binding site in 3′-UTR of *AID*, an increase of class switch recombination and defective affinity maturation is observed [49]. Notably, a substantial genomic instability is also found, characterised by a high degree of *MYC*-Igh related translocations that are well known as transformation events in Burkitt’s lymphoma [74]. This notable finding is in accordance with the similar but more pronounced genomic instability and defective immune response phenotype observed in miR-155-deficient mice, supporting the belief that physiological levels of miR-155 expression are not only crucial for B-cell functions, but also exert a protective role against potentially oncogenic *MYC*-related injuries. However, the absence of any B-cell neoplasm in miR-155 deficient and in *AID* -mutant mice indicates that this mechanism itself is not sufficient to induce malignancy, thereby suggesting the presence of other systems which protect from *AID*-related malignant transformation. The regulation of somatic hypermutation and class-switch recombination carried out by miR-155 seems to be shared with miR-181b that too targets *AID*. In particular, these two miRNAs together regulated the levels of *AID* expression at different stages of B-cell activation [49]. Finally, miR-155 also targets *PU.1*, another important transcription factor regulating the activation and function of B cells. Okada *et al.* referred that following miR-155 deletion in mice, *PU.1* resulted over-expressed allowing a defective generation of IgG1 switched cells as the result of deregulation of immunoglobulin switching [63]. Recently Rai *et al.* [65] demonstrated that miR-155 directly targeted the transcriptional factor *Bone Morphogenetic Protein* (*BMP*)*-responsive transcriptional factor* (*SMAD5*). Conversely, this transcriptional factor is activated by *Transforming Growth Factor Beta1* (*TGF-β1*). Thus in DLBCL the over expression of miR-155 inhibits *SMAD5*, which classically acts in association with signals transduced by the *BMP* family of cytokines [99]. Accordingly, the disruption of the *SMAD5* activity in this malignancy prevents the TGF-β growth-inhibitory effects [65]. This finding highlight the mechanism trough miR-155 regulates the death and the survival of B-cells. In accordance with this Pedersen *et al.* [67] demonstrated that miR-155 targeted *Src Homology-2 domain-containing Inositol 5-Phosphatase 1* (*INPP5D*) (also known as SHIP1) that antagonizes the *Phosphatidylinositol 3-Kinase* (PI3K) signaling from which strongly depends B-cell survival and fate determination [68,69,70]. Finally, more recently Dagan *et al.* [100] demonstrated that miR-155 directly down-regulates *Human Germinal center–Associated Lymphoma* (*HGAL*) expression by binding to its 3'-untranslated region. *HGAL* is a protein specifically expressed in the cytoplasm of germinal center (GC) B-cells, but absent in mantle and marginal zone B-cells and in the interfollicular and paracortical regions in normal tonsils and lymph nodes [64]. Accordingly it is a specific marker of GC-derived lymphomas such as DLBCL in which it exhibit prognostic significance as well as in cHL [64]. Physiologically this protein inhibits lymphocyte and lymphoma cell motility by activating on the RhoA signaling cascade and interacting with actin and myosin proteins [101]. However little is known about its regulation. Recently it was demonstrated that *HGAL* was specifically targeted by miR-155 leading to a decreasing of RhoA activation and increasing of spontaneous and chemoattractant-induced lymphoma cell motility [100]. Through this repression function, miR-155 may be involved in the loss of *HGAL* expression on differentiation of human GC B cells to plasma cells. Furthermore, the effect on lymphoma cell mobility may partially contribute to lymphoma dissemination and aggressiveness, typically observed in DLBCL patients that express high levels of miR-155 and lack *HGAL* expression [100].

As previously mentioned, one the most important steps of lymphopoiesis depends on the cross-talk between lymphoid and other immune cells and regulated by miRNAs is the terminal differentiation process into plasma cells and memory B-cells. This process is strictly dependent on the close contact-cell direct interaction between FDCs and B-cells. It is also strongly regulated by the suppression or induction of specific transcription factors required for the survival signal to protect B-cells from apoptosis and essential for their maturation. Indeed, Lin *et al.* [85] recently demonstrated that by binding to B-cells of GC, FDCs modulated in a cell-cell contact dependent manner their miRNA expression profile by inducing specific down-regulation of the miR-9 and Let-7 families and up-regulation of miR-30. This in turn resulted in, respectively, the up-regulation of the *PR domain containing protein 1* (*PRDM1*) expression (also know as blimp-1) and the down-regulation of *B-cell lymphoma 6* (*BCL6*) expressions [85,86]. The *PRDM1* is a key differentiation factor in post-germinal center (GC) cells and it is regarded as a master regulator for plasma cell differentiation. Microarray profiling demonstrates that *PRDM1/Blimp-1* orchestrates plasma cell differentiation by repressing genetic programs associated with activated B cells and/or GC B cells, including those that control cell proliferation, and by activating genetic programs associated with plasma cell functions, including apoptosis [86]. Specifically, the over expression of *PRDM1* is crucial for B cell terminal differentiation into plasma cells and memory B-cells, while the silencing of *BCL6* is important for determining the ability of developing B-cells to move to the GC. Conversely, reduced expression of *PRDM1* prevents plasma cell differentiation from B cells originating in the GC. The *PRDM1/blimp-1* is a target for miRNA-mediated down-regulation by miR-9 and let-7a [86]. Therefore, the balance between *BCL6* and *PRDM1* by controlling miR-9 and Let-7 (as well as miR-30) expression represents a significant regulatory mechanism of B-cell differentiation. Dysregulation of this mechanism may interfere with B-cell survival and maturation, suggesting its potential involvement in the pathogenesis of B-cell lymphomas [85,86,88,102,103]. As confirmation, the *PRDM1/blimp1* locus lies on chromosome 6q21-q22.1, a region frequently deleted in B cell lymphomas, suggesting that it may harbour a tumor suppressor gene. In addition, some studies supported a role for interference of *PRDM1* functions in the pathogenesis of human lymphomas. They showed that *PRDM1* was inactivated by a classic mechanism for tumor suppressor genes in non-GCB-DLBCL, strongly supporting that inhibition of post-GC differentiation of B-cells toward plasma cells may play a role in lymphoma pathogenesis [88,103].

## 5. The Role of miRNAs in T-Cell Maturation

**Figure 3:**
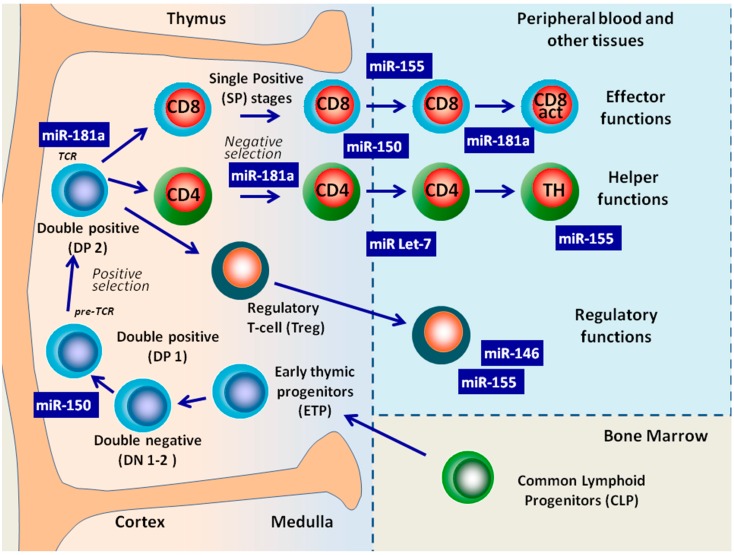
MiRNAs Involved in the regulation of T cell development from common lymphoid progenitor cell to produce functionally mature T lymphocytes.

The development of T-cells takes place in the thymus through a complex signaling network that regulates the transition of T-cell progenitors in pro-T and pre-T thymocytes. Subsequently pre-thymocytes progress through well-defined stages that, based on CD4 and CD8 expression, can be subdivided into double-negative (DN), double-positive (DP), and single-positive (SP) CD4+ or CD8+ thymocytes [23,45]. During this differentiation process T-cell progenitors undergo rearrangements of T-cell receptor (*TCR*) genes, alternating phases of intense selection with phases of proliferation (Figure 3). The ontogenesis of T-cells in the thymus has not yet been clearly defined molecularly; however, when comparing the miRNA expression profiles of the DP, SP CD4+ and SP CD8+ of human thymocytes, distinct microRNA expression profiles that reflected the developmental stages were observed. In particular, the dynamic nature of miRNA gene transcription and processing through the developmental process was revealed [23,45,104,105].

### 5.1. T-Cell Development in the Absence of Dicer

The activity of Dicer on the development and function of T-cells has been investigated in mice through its conditional deletion at different stages of differentiation, e.g., early T-cells, DP thymocytes, T effector cells, and T regulatory cells (Treg). Dicer deletion early in T-cell development (DN3 stage) impaired the survival of αβ T lineage cells, while paradoxically the number of γδ expressing thymocytes did not change. This suggested that Dicer-dependent mechanisms possibly controlled αβ/γδ lineage choice directly. Furthermore, the number of both more advanced stage thymocytes (DP, SP) and peripheral T-cells underwent a significant reduction, most likely due to the increase of cellular apoptosis. This strongly suggested that the whole T lineage development was compromised. However, the percentages of the different subsets of the thymocytes remained unchanged as compared to normal controls [106]. Therefore, while Dicer appeared to be essential for the growth and survival of developing T DN progenitors, it exerted a dispensable role on specific T lineage differentiation, possibly due to the well-known redundancy of transcription factors regulating T differentiation at the DN stage. Differently, conditional deletion of Dicer using a Cre transgene driven by the CD4 enhancer/promoter/silencer (CD4cre) in mouse DP thymocytes, the major T-cell subset in the thymus [107], resulted in moderate reduction of CD4+ T cells as well as severe reduction of peripheral CD8+ T cell compartment, and specific alterations of both T helper (Th) cell differentiation and cytokine production [108]. Although Dicer-deficient CD4 + T cells were reduced in number, residual cells were viable and were therefore analysed further. These cells exhibited defects in microRNA processing, proliferated poorly, and underwent increased apoptosis in response to *in vitro* stimulation. In contrast generation of B220+ mature B-cells was unaffected in the mutants [108]. Interestingly, the Dicer deficient Th cells, which also presented a defect in proliferation, preferentially produced interferon-gamma, the hallmark effector cytokine of the Th1 lineage, retaining their ability to produce IL-4, which is typically secreted by Th2 cells. Accordingly, Dicer was not essential for the lineage choice between the Th1 and Th2 cells. Nevertheless, *in vivo* study showed an opposite observation with a preferential bias towards Th1 differentiation upon Dicer deletion, thus suggesting that Dicer is required to repress the Th1 genetic program [108]. Tian *et al.* [109] showed the impact of miRNA loss on T cell activation, effector T cell differentiation and autoimmune disease by removing Dicer from the T cells of both wild type mice and TGF-β receptor-deficient mice. They observed a potent suppression of T cell activation, INF-γ production and autoimmune disease in all targeted organs except the colon, in which miRNAs demonstrated tolerogenic function. These findings demonstrated that the loss of T-cell miRNA provides systemic protection against autoimmune disease in mice thus reversing the original conclusion of miRNA function in T cells by revealing a systemic pro-autoimmune function [109]. Significantly, proper regulation of Th1/Th2 lineage decisions along with cytokine gene activation and silencing has been found to be crucial for the effective immune function and the prevention of autoimmunity or allergy [110].

Finally, a notable effect of Dicer deletion in DP stage is the decreased proportion of transcription factor *Forkhead box P3* (*FOXP3*)+ regulatory T cells, with a reduction of their number in the thymus, spleen and lymph nodes, suggesting that Treg cell development involves Dicer-generated RNAs [111].

Likewise, in the periphery the Dicer-dependent miRNA pathway is essential for the generation and function of Treg cells. This has been confirmed in a study in which the lineage-specific deletion of Dicer in *FOXP3*-Cre BAC transgenic or *FOXP3*-Cre knock-in mouse strains greatly reduced the suppressive capacity of Treg cells. However, minimal changes in their number and proliferation were detected in the peripheral compartments [112,113,114]. Notably, under inflammatory conditions this severe Treg cell dysfunction led to a high morbidity and mortality due to a lymphoproliferative autoimmune syndrome with an early onset. These effects were much more dramatic as compared with the late-onset mild autoimmune disease caused by the deletion of Dicer in the DN/DP stages. This is because the great reduction in the number of peripheral Treg and the dysfunction of their suppressive activity were probably compensated by the concomitant reduction of effector cells and/or by residual normal Foxp3+ cells [108].

In another subset of regulatory lymphocytes, the invariant natural killer T (iNKT) cells, a substantial reduction in number along with severe dysfunction has been observed in both the thymus and peripheral lymphoid organs of Dicer knockout mice [115]. Like conventional T-cells, iNKT cells originate from DP thymocytes as a small population of T-lymphocytes expressing surface markers characteristic of both T cells and natural killer (NK) cells. Selection and lineage fate at this stage depends on TCR signal strength. Zietara *et al.* [116] demonstrated the critical role of the miR-181a/b cluster in iNKT cell development through the modulation of TCR signaling threshold for agonist selection. The deletion of this cluster reduced the responsiveness of DP thymocytes to TCR signals and virtually abrogated early iNKT cell development, resulting in a dramatic reduction in iNKT cell numbers in thymus as well as in the periphery. Conversely, increased concentrations of agonist ligand recovering iNKT cell development in miR-181a/b-1^−/−^ mice [116]. Similarly, Henao-Mejia *et al.* [117] observed in miR-181-deficient mice a complete absence of mature NKT cells in the thymus and periphery as well as a severe defect in lymphoid development and T-cell homeostasis associated with impaired PI3K signaling. This suggested that miR-181 modulated expression of the phosphatase *PTEN* to control PI3K signaling, which was a primary stimulus for anabolic metabolism in immune cells [117]. As an important link between the innate and adaptive immune responses, the iNKT cells play important roles in immune surveillance, including tumor surveillance. In particular, they control and activate NK cells, a different population of effector/regulatory cells. The development, maturation and functions of these population are also affected by Dicer deletion induced in BM CLP [115].

Collectively, the preponderance of evidence from Dicer knockout models suggests that miRNAs regulate the cellular survival, maturation, and function of the different stages of T-cell development, but the effects of the total elimination of miRNAs are difficult to extricate from those of individual miRNAs and/or overlapping effects of other Dicer targets. To address these critical questions, many research teams have carried out studies on several individual miRNAs. Among these, miR-181a, miR-146, miR-155, let7 and miR-15 have mostly been identified as key regulators of T cell development and homeostasis (Figure 3 and Table 2).

### 5.2. Individual MiRNAs Have the Potential to Regulate T-Cell Development and Functions

Intrathymic T-cell development is strongly regulated by miR-181, which is highly expressed in the thymus [19], the primary lymphoid organ containing mainly T-lymphocytes. Neilson *et al.* [45] showed that in this lymphoid organ miR-181 is transiently up-regulated in the CD4+/CD8+ DP thymocytes, while in SP and mature T-cells its expression is very low. The suggested role of miR-181 in T-cell development is the government of the CD4/CD8 lymphocyte homeostasis by selectively regulating the levels of CD69, *BCL2* and *T-cell receptor* (*TCR*)-α. All these molecules are important for positive selection, and they are normally expressed at high levels in DP thymocytes. CD69 is further involved in the generation and normal migration of SP thymocytes from the thymus [19,115]. Taken together, the above findings indicate that miR-181 affects T-cell selection and the levels of circulating T-lymphocytes through the following mechanisms: (a) it controls the ability of the CD4 or CD8 lymphocytes that express CD69 to leave the thymus by modulating the CD69 signaling pathways; (b) it induces the cell death of DP thymocytes by targeting *BCL*2 upon positive selection; and (c) it shifts the threshold for positive and negative selection by reducing the levels of *TCR*-α expression. All these mechanisms support the role of miR-181 in the late thymic stages of T-cell development [118] and suggest a potential involvement of miR-181 in T lymphoma pathogenesis mainly through an impaired control of *BCL2* expression as well as of T-cell receptor (TCR) sensitivity acquisition. Particularly, the latter is properly regarded as a crucial step of T-cell development. During the late stages of maturation, T-cells acquire the ability to generate effector mechanisms aimed at eliminating antigens. The acquisition of functional maturation requires the expression on the surface or within the cell of molecules, involved in the process that leads to the activation and function of effector lymphocytes. However, studies carried out on the miRNA expression profile revealed that very few miRNAs were dynamically expressed during the acquisition of the molecules related to the different steps of T-cell differentiation [104,119]. The control of TCR sensitivity to peptide antigens in mature T-lymphocytes is mediated by miR-181, which modulates TCR signaling strength and sensitivities most likely through the down-regulation of several protein tyrosine phosphatases, including SHP2, PTPN22, DUSP5 and DUSP6, which in turn usually damp the TCR signaling threshold by targeting *lymphocyte-specific protein tyrosine kinase* (LcK) and *Extracellular signal-regulated kinases* (Erk). Accordingly, the up-regulation of miR-181 in mature T-lymphocytes was related to a higher sensitivity to peptide antigens, while its inhibition in immature lymphoid T-cells both decreased the sensitivity and impaired the selection of T-cells, following respectively the paucity/accumulation of the above phosphatases [118].

Upon activation, antigen-specific naive T-cells undergo a programmed fate that separates them into two subsets determined by the expression of the receptors CC-chemokine receptor 7 (CCR7) and L-selectin (CD62L), two receptors required for migration to secondary lymphoid organs. This distinction is important since CCR7-CD62high “effector” memory T-cells provide immediate protection against pathogens, whereas CCR7+CD62low “central” memory T-cells play a role in long term protection as they can proliferate and become effector cells upon secondary stimulation with antigen. The decision to differentiate preferentially into central memory T-cells is another important level of the regulation of miRNA functions. Indeed, using an *in vitro* system in which CD8 T-cells are activated in order to induce differentiation into the memory or effector phenotypes, it was recently observed that this differentiation process depends on the balance of the effects of a discrete number of miRNAs including let-7 family members, miR-155, and miR-150 [59].

With regard to CD4+ effector T-cells, also called helper T (Th) cells, it was recently demonstrated that miR-150, which is up-regulated in resting naive CD4+ T cells and down-regulated during their further differentiation into Th1 and Th2 cells [50], represents one of the top up-regulated miRNAs involved in normal human T-cell differentiation. This has been suggested by comparing the miRNA expression profiles of human thymocytes at different stages of maturation [120]. In particular, Ghisi *et al.* [45] showed that miR-150 regulated the differentiation from DP into CD4+ and CD8+ T-cells by *NOTCH3*, a new potential target of this miRNA. *NOTCH3* is a member of the Notch receptor family, which plays an important role in both T-cell differentiation and leukaemogenesis [121,122] by controlling T-cell survival, proliferation and differentiation as well as neoplastic transformation. The existence of a miR-150/*NOTCH3* pathway was supported by indirect evidence of their inverse expression patterns, as *NOTCH3* expression levels were significantly higher in DN and DP thymocytes than in mature SP cells. This is opposite to the pattern of miR-150. In addition, the ectopic expression of miR-150 in T-cell progenitors reduced *NOTCH3* levels in T-cell lines, and interfered with the transition from CD44−CD25+ DN3 to CD44−CD25−DN4, thus depressing proliferation and survival signals [18]. Finally, two studies have shown that *NOTCH3* plays a crucial role in the intrathymic pre T-cell receptor selection of T-cells [122,123]. The control of the *NOTCH3* pathway through miR-150 could therefore have an important impact on T-cell development, suggesting a potential involvement in T-cell lymphomagenesis when dysregulated.

The Th lineage decisions are also specifically regulated by miR-155 control. This miRNA favours Th1 responses partially by modulating cytokine production; in mice models miR-155 deletion led to a bias toward Th2 differentiation along with an impaired Th17, and Th1 generation. The above findings were linked to the incapacity of miR-155 deleted cells to produce significant levels of interleukin-2 (IL-2), interleukin-17 and interferon-γ (INF-γ) [60,61]. The involvement of miR-155 in inflammatory responses and autoimmunity is also supported by the reported high resistance of miR-155^−/−^ mice to experimental autoimmune encephalomyelitis (EAE) as a consequence of impaired Th17 and Th1 differentiation [124]. Notably, miR-155 also controls the development and homeostasis of Treg cells by stabilizing the signal of transcription factor *FOXP3* through the down-expression of an important negative regulator of IL-2, the *suppressor of cytokine signaling 1* (*SOCS1*). In turn, *FOXP3* up-regulates the expression of miR-155 [124]. The end result of this reciprocal *FOXP3*/miR-155 interaction confers on Tregs the propensity for proliferation in a competitive environment, without inducing significant changes in their suppressor activities. The above-mentioned studies and many others that we have not been mentioned here highlight that miR-155 can regulate different aspects of the immune response, representing the connection between inflammation, immunity and cancer. More specifically, miR-155 is involved, mostly as a pro-inflammatory molecule, in innate immune response by up-regulating *Tumor Necrosis Factor alpha* (TNF-α) in LPS-activated monocytes [125]. It is also involved in the adaptive immune system where it promotes inflammatory responses by enhancing Th1 generation as well as contributing, as previously mentioned, to humoral responses by regulating the function of DCs and the antibody repertoire [60,61]. It must be noted however that the effects of this miRNA are dependent on the cellular context, given that miR-155 plays negative regulatory roles in immune responses [126,127].

MiR-146 also provides regulation of Treg cells by ensuring their suppressor functions, mainly when exposed to an inflammatory environment [128]. Like miR-155, this miRNA is highly expressed in Treg cells and is induced upon activation of effector T-cells. Loss of miR-146a in Treg cells has been associated to an increase in their number in mice along with a severe impairment of their suppressor activity, leading to a fatal Th1-mediated disease as a result of an immunological tolerance breakdown due to dysregulated IFNγ responses. This was likely due to the increased expression and activation of *Signal transducer and activator transcription 1 (STAT1),* a direct target of miR-146a [45]. Likewise, increased Stat1 activation in Treg cells lacking selectively *SOCS1*, a key negative regulator of IFNγ-induced phosphorylation of *STAT1*, has been associated with a similar Th1-mediated disease. Accordingly, these results suggest that miR-146 controls specific aspects of the Treg suppressor function by modulating IFNγ responses through up-regulation and activation. In line with this, an optimal range of Stat1 activation appears essential for Treg-mediated control of Th1 responses in autoimmune or other diseases [45]. As well as Treg regulation, miR-146 could be more generally involved in T-cell differentiation as its expression substantially increases in Th1 cells and decreases in Th2 cells as compared with naive T-cells [45,50].

**Table 2:** The main miRNAs involved in the different stages of T cell development.

miRNAs	Target	Function
miR-181a	CD69 *BCL2* TCR-α (DUSP5, DSP6, SHP2, PTPN22)	Regulation of positive selection by governing the homeostasis of CD4/CD8 lymphocytes and modulation of T-cell sensitivity by increasing TCR signaling to peptide antigens through the down-regulation of multiple phosphatases [19,23,45,104,118,126]. Regulation of iNKT cell development through the modulation of TCR signaling threshold resulting the increase responsiveness of DP thymocytes to TCR signals [116].
miR-17-92	*CREB1* *PTEN* Bim	Regulation of effector and memory CD8+ T-cell differentiation. Temporal expression is critical [129].
miR-150	*NOTCH3*	Controls of T-cell differentiation [51]. Regulation of differentiation into the memory or effector phenotypes of T cells [59]. Regulation of the differentiation from DP into CD4+ and CD8+ T-cells [45,120,121,122]. Regulation of the intrathymic pre-T-cell receptor selection of T-cells [121,123]
miR-155	*SOCS1*	Regulation of differentiation into the memory or effector phenotypes of T cells [59]. Controls of T-cell differentiation: to favour Th1 responses partially by modulating cytokine production [60,61]. Control of proliferation and homeostasis of Treg cells by stabilizing the signal of *FOXP3* through the targeting of *SOCS1* [124].
miR-146	*STAT1*	Regulation of the Treg suppressor function by modulating IFNγ responses through the targeting of *STAT1* [45,128]: promotion of differentiation into Th1 cells rather than Th2 cells [45,50]

## 6. miRNAs and Lymphoid Malignancies

Lymphomas are a heterogeneous group of lymphoproliferative disorders, primarily divided into Hodgkin (HL) and non-Hodgkin lymphomas (NHL). They originate from the lymphocytes of almost all stages of the B- and T lineage in response to abnormalities in the molecular mechanisms that regulate lineage-specific differentiation. Like most cancers, the natural course of lymphoma is characterized by tumor progression, marked by a stream of events leading to the enhancement of proliferative and invasive ability towards the establishment of a more aggressive phenotype. Although the specific determinants of the pathogenesis, natural history and evolution of lymphomas are still largely unknown, an ever-increasing amount of evidence suggests that a leading role is played by miRNAs. As we have described above, miRNAs are directly or indirectly involved in cell-survival, cell death, proliferation and differentiation. Thus, it is not surprising that dysregulated miRNA expression has now been documented in a broad range of tumors, including almost all types of studied lymphomas, and that abnormalities in certain miRNAs play a potentially causal role in lymphomas. In addition, recent findings indicate that alterations in the expression of several miRNAs are often present in human cancers and that more than 50% of human miRNA genes are located in common breakpoint cancer- and lymphoma-associated regions genetically altered or in fragile sites [130,131,132]. A direct causative link between a lymphoproliferative disorder and miRNA was first revealed in 2002 by Calin *et al.* [133] who demonstrated that two miRNAs clustered in chromosomal region 13q14, miR-15a and miR-16-1, were frequently deleted or down-regulated in B-CLL. Given that it was known that the loss of chromosomal region 13q14 was strongly associated with B-CLL, these findings suggested that non-coding genes were contributing to the development of cancer, and paved the way for a closer investigation of miRNA loss or amplification in tumours.

MiRNAs not only virtually control the development and proliferation of lymphoid cells but also directly participate in the regulation of all immune-related processes by modulating the interactions with the environment. Conversely the activity of miRNAs is influenced by master regulators (such as c*-MYC* or *TP53*) which respond to the cellular context and environmental stimuli to possibly maintain a dynamic equilibrium between the cell and the environment. Any unbalance of this equilibrium could lead to aberrant expression/function of miRNAs, which contributes to the pathogenesis and progression of lymphomas. Moreover, since the miRNA profiles reflect the developmental lineage and differentiation state of many tumors, including lymphoid malignancies, these profiles can be used to classify poorly differentiated tumors [134,135].

The potential function of miRNAs in lymphoma formation and progression has been studied by detecting their expression profiles in lymphoid tissues and by evaluating the consequences of the up- and/or down-regulated expression of candidate miRNAs. This was carried out using several methods, such as antisense inhibitors to block the targeted miRNA function as well as transgenic mutants that knockdown or overexpress specific miRNAs. The knowledge of the physiological functions related to a specific miRNA allows us to understand its role and biological effects in tumor pathogenesis. This highlights that the same miRNA may participate in cancer prevention/generation by directly/indirectly regulating cell growth and/or controlling apoptosis [136] through the modulation of different transcription factors or signaling pathways depending on the cellular context and the target genes. In the complex environment of immune responses and cell differentiation, miRNAs may act as potential oncogenes (oncomirs) or as tumor suppressor genes by interacting with regulatory networks of oncogenes and/or tumor suppressor genes. The central role of these interactions and the importance of miRNAs in tumor pathogenesis are confirmed when regulatory functions are lost following their dysregulation. Such is the case of lymphoid malignancies in which the most important targets/actors of the regulatory networks (e.g., c*-MYC*, *MYB*, *PTEN*, *TP53*, *BCL6*, *BCL2*, *RB2*, *BIC* and *RAS*) are frequently dysregulated concomitantly with the aberrant expression of some specific miRNAs which are known for their potential to act as oncogenes or tumor suppressor genes in haematological tissues. Notably, the same miRNA may be a tumor suppressor or oncogene depending on the cellular context in which it is expressed. This means that defining its precise contribution to lymphomagenesis can be challenging (Table 3).

### 6.1. miRNAs as Oncomirs in Lymphoid Malignancies

Some miRNAs may induce the expression of genes that promote cell proliferation and survival, as well as inhibit the expression of tumor suppressor genes and/or genes that control cell death. Accordingly these miRNAs are considered oncogenes, also called “*oncomirs*”, and thus are usually down-regulated in physiological conditions. Conversely the increase of their expression, as detected in several tumors, induces up-regulation of the genes promoting cell growth and/or dampens the tumor suppressor genes and/or genes that control apoptosis. All of this promotes neoplasms, or in some cases, evident malignancies. Several miRNAs such as miR-155 and miR-17-92 cluster are known as oncomirs and are increased or aberrantly expressed in lymphomas.

MiR-155 is one of the most intensely studied miRNAs. This is because it is abnormally expressed in HL as well as in a wide range of B-cell lymphomas, the most common form of NHL, and especially in the aggressive subtypes such as diffuse B large cell lymphoma (DBLCL) and mantle cell lymphoma (MCL). A large body of research, some also mentioned, supports the key role of this miRNA in lymphoid malignancy development. Fulci *et al.* [137] assessed the miRNA expression profile in B-CLL cells using two independent quantitative methods, showing that miR-155 was on average 5.3-fold with a maximum of up to 23-fold up-regulated in B-CLL as compared to a pool of normal controls. This finding supports the hypothesis that the over expression of miR-155 could play an important role in promoting B-lymphocyte proliferation. Moreover, as no amplification of the genomic locus encoding this miRNA was revealed by this study, the dysregulation of miR-155 is likely to occur at the transcriptional or posttranscriptional level in B-CLL [137]. Eis *et al.* [71] reported 10 to 60 fold increases in the levels of miR-155 in DBLCL cells as compared to normal circulating B-cells. Interestingly they found significantly higher levels of miR-155 in the activated B-cell-like (ABC-DBLCL) phenotype than in the germinal centre B-cell-like (GC-DBLCL) phenotype, suggesting that miR-155 may be useful for differential diagnosis of ABC-DBLCL patients and as a prognostic factor considering the worse clinical prognosis of these patients as compared with GC-DBLCL [71,138]. Recently high serum levels of miR-155 have been shown in DBLCL patients as compared with normal controls, demonstrating that it can predict DBLCL with a sensitivity of 83% and a specificity of 65% [139]. Accordingly miR-155 could be a potentially useful tool as a novel non-invasive biomarker for the diagnosis of DBLCL. Principally, three mechanisms involving *PU.1, INPP5D* and *SMAD5* pathways were identified to explain the contribution of miR-155 to DBLCL development.

Firstly, considering the aforementioned functions of miR-155 in B-lymphocyte development [60,61] and especially the nature of GC B-cell responses which involve the *PU.1* pathway, it is plausible that miR-155 may directly down-regulate the expression of its target *PU.1* [63], which is a member of the *E26 transformation-specific* (*ETS*) domain-transcription factor family required for later stages of B-cell differentiation and has tumor suppressor activity in B-cells [73]. In several lymphoma types the absence or low expression levels of *PU.1* are associated with constitutive activation of *Nuclear Factor kappa-light-chain-enhancer of activated B-cells* (NF-κB), a transcription factors which is both causally connected to lymphoma development and determines poor clinical outcome when aberrantly activated. Interestingly, it has been shown that the high expression of CD10, a specific key marker of GC-DBLCL, is conversely down-regulated or absent in ABC-DBLCL cells in which there is simultaneously the aberrantly constitutive activation of the NF-κB pathway and the constitutive overexpression of miR-155 with low expression levels of PU.1 [140]. In line with these findings, Rai *et al.* [66] described the correlation between miR-155 expression and the constitutive expression of NF-κB both in DBLCL cell lines and ABC-DBLCL primary cells. Therefore, at least in some more aggressive B-cell lymphomas it is likely that pathogenic aberrant activation of NF-κB can induce increased expression of miR-155, which then down-regulates *PU.1*, and consequently leads to reduce CD10 expression [140].

Secondly, the PI3K/AKT pathway is one of the most potent pro-survival signaling cascades. It is aberrantly activated in a variety of lymphomas and considered an important unfavourable prognostic factor. Several studies have suggested that miR-155 regulates this pathway by directly targeting the expression of *INPP5D*, a negative regulator of the PI3K component of PI3K/AKT [140,141,142]. Therefore *INPP5D* may represent the molecular link between the overexpression of miR-155 and the activation of PI3K-AKT in DBLCL [142]. Accordingly, in both *in vitro* and *in vivo* xenotransplant models the abnormal growth of DBLCL cells depended on the lack of negative signals on cell survival and proliferation consequent to the suppression of *INPP5D* expression. This is due to the rise of miR-155 levels induced in turn via autocrine stimulation by TNF-α rather than by genomic mutations [67]. Intriguingly anti- TNF-α drugs (eternacept or infliximab) were able to reverse these effects leading to the reduction of miR-155 levels and the restoration of *INPP5D* expression in DLBCL cells *in vitro*, and to the subsequent inhibition of cell proliferation and tumour growth *in vivo* [67]. Other support for the plausibility and relevance of *INPP5D*-related mechanisms comes from the observation that the levels of *INPP5D* are expressed differentially between ABC-DLBCL and GC-DBLCL [68], and significant differences in miR-155 expression levels between these two subtypes of DLBCL have been reported [72,138]. These findings provide insight that the miR-155/*INPP5D* pattern may also be a potential diagnostic marker. As the assessment of both the miR-155/*INPP5D* and miR-155/*PU.1* axes may potentially be useful for differential lymphoma diagnosis between ABC-DBLCL and GC-DBLCL, this concurs with the concept that different networks are involved in the development of different lymphoma subtypes, thus reflecting their origin and particular stage of differentiation.

Through the third mechanism, miR-155 specifically targets and suppresses the expression of the transcriptional factor *SMAD5* that is non-canonically activated in cell lines and primary cells of DBLCL by TGF-beta1 signaling. When appropriate this is an essential homeostatic regulator of normal cellular processes, while when altered it promotes leukaemogenesis and lymphomagenesis. Like TGF-beta1, *BMP* is a member of the TGF-beta super family, and both demonstrate functions as tumor suppressors. Since the overexpression of miR-155 by targeting *SMAD5* makes DBLCL cells resistant to the growth-inhibitory effects of both TGF-beta1 and *BMP, SMAD5* may represent a unique mechanism used by lymphoma cells to escape the tumor suppressive effects of the TGF-beta family [65]. This also makes miR-155 a potential target for therapeutic intervention.

MiR-155 and (*BIC*), a primary transcript of miR-155, are highly expressed in HL. On the contrary, they are unexpressed in the neoplastic cells of most NHL subtypes with the exception of DBLCL, primary mediastinal B cell lymphoma (PMBL) and paediatric Burkitt's lymphoma (BL). This suggests that it is a specific biomarker of HL [143,144,145]. BIC activation is associated with many cancers, but most important it may accelerate the development of lymphoma and leukaemia. This finding suggests that BIC plays a role as a proto-oncogene, and its activity [71] may depend on miR-155 since this miRNA is located in the only phylogenetically conserved region of BIC [146,147]. Finally, miR-155 is highly expressed in indolent lymphoma such as B-CLL and marginal zone lymphoma (MZL) [71]. Surprisingly, in contrast to DBLCL and HL, adult BL cells are characterized by down-regulation of miR-155 [75], although conversely high levels of precursor miR-155/BIC RNA have been found in paediatric BL [148]. BL pathogenesis is strongly associated with the Epstein-Barr virus (EBV) infection, and recently it the interplay between EBV, miRNAs and BL has been described. In particular a differentiated expression of BIC and miR-155 in three latency type II EBV-positive BL cell lines and in all primary post-transplantation EBV-related lymphoproliferative disorder cases has been shown, suggesting that the activation of BIC expression is linked to viral infections. This hypothesis seems also to be supported by the observation that dysregulation of miR-155 has been found not only in lymphoma-associated EBV, but also in Kaposi sarcoma associated herpesvirus (KSHV) and Marek disease virus (MDV); a persistent infection of lymphocytes induced through the activation of latent membrane protein 1 (LMP1) by EBV [149] and the production of viral miR-155 orthologous by KSHV [150] and MDV [151] has been demonstrated. These findings suggest that the aetiological starting point of virus-associated lymphoma is the continuous stimulation of lymphocytes by miR-155 or its viral orthologues. Finally, the differences in miR-155 expression between paediatric and adult BL probably indicate a specific-age-dependent role of this miRNA, and are also an example of how the same miRNA can have bivalent roles in cancer. In fact, given that miR-155 has pleiotropic effects in different cell lineages by targeting different mRNAs, the aberrant expression of this miRNA, as is probably the case with other miRNAs, contributes to the development of lymphoid malignancies. This is based on the type of cell/stage or, alternatively, on how changes in the microenvironment cues modulate the effects of miR-155 aberrant expression.

Another very important oncomir is the miR-17-92 cluster, located at chromosome 13q31-q32, a region frequently amplified in lymphomas and especially in aggressive B-cell lymphoma such as GC-DBLCL [31,40] and MCL [37]. It has been hypothesized that its oncogenic effects mainly depend on the dysregulation of Bim, *PTEN* and the *MYC/E2F1* loop. In particular, it has been suggested that the effects of miR-17-92 over expression on the onset of lymphoma are due to the constitutive down-regulation of PTEN and Bim, two tumor suppressor proteins with respectively anti-proliferative and pro-apoptotic activities that control B-cell development. Olive *et al.* [36] demonstrated that miR-17-92 activates the AKT1/mTOR pathway by functionally antagonizing PTEN to promote cell survival. It follows an increased proliferation and a decreased activation of cell-death, which gives rise to an aberrant growth of B-cells and consequently to the onset of lymphoma [29,30]. Another pathogenic mechanism that may operate in miR-17-92-dependent lymphomas is based on the associations between miR-17-92 over expression and translocations of the *MYC* gene, an oncogene that is a critical mediator of cell growth.

O’Donnell *et al.* [33] have documented the directly activation of miR-17-92 by the c-*MYC* oncogene. In addition to c-*MYC*, the transcription of miR-17-92 is directly activated by *E2F1* and *E2F3*. The last one is the major E2F family member that binds to the mir-17-92 promoter region [34,39]. *E2Fs* are essential for the progression of the cell cycle, activating a large number of S phase genes. These finding are consistent with the functions of mir-17-92 in promoting proliferation in a variety of cell types such as lymphoid. Consequently, the cycling cells are likely to have elevated miR-17-92 due to the periodic burst of *E2F* activity during S phase, while quiescent cells may have reduced miR-17-92 levels. The high frequency of association suggests that *MYC* aberrant signaling up-regulates miR-17-92, that supported by *E2F* activity, induces B-cell hyperproliferation not appropriately balanced by apoptosis [34]. It is likely that the synergistic effects of the *MYC*/miR-17-92/*E2F* circuit, generated from *MYC* translocations, accelerate the development of lymphoid malignancies as well as increase their aggressiveness [35].

Recently it has been demonstrated that the miR-17-92 cluster mediates chemo- and radio-resistance, as well as enhances tumor growth in MCL by activating the PI3K/AKT pathway trough the suppression of the *PH domain and Leucine rich repeat Protein Phosphatases* (*PHLPP2*) an important negative regulator of the PI3K/AKT pathway [37,152] This therefore suggests that another mechanism of action mediated by the miR-17-92 cluster is to promote cell growth by targeting *PHLPP2* in addition to *PTEN*, Bim and *MYC/E2F1* [37]. Furthermore, these studies highlighted the potentially of miRNAs to influence the tumor development not only trough the onset but also compromising the response to therapy.

One of the first miRNAs to be discovered in human cells, miR-21 is overexpressed in most cancer types and haematological malignancies analysed so far, including both LH and LNH [42,138]. Although miR-21 was initially noted as an apoptotic suppressor in various cell lines, in subsequent studies it was established as a ubiquitous anti-apoptotic oncomir.

Several studies have addressed the significance of miR-21 expression as a prognostic marker in DBLCL, but have yielded variable results. Initial studies showed that high levels of miR-21 expression in biopsy material were associated with a better relapse-free survival in 35 patients with de novo DBLCL, mostly treated without anti-CD20 MoAb [138]. Importantly, its expression was reported as a significant prognostic indicator, independently of the International Prognostic Index (IPI) and other clinical pathological characteristics. These findings appeared consistent with the observation that miR-21, along with miR-155, was found to be more highly expressed in the poor risk ABC subtype than in the GC-DBLCL subtype [138]. However, the prognostic value of mir-21 was not confirmed in more recent studies, which used high resolution profiling in combined integrated models of miRNAs in patients treated with R-CHOP [137]. All the above results should however be interpreted with caution and viewed in the light of many limitations such as the retrospective nature of the studies, different treatments, and the diverse characteristics of the study populations as well as the discrepancy in both method and tissues used by different research groups to assess miRNA expression. Notably, elevated circulating miR-21 has been associated with improved relapse-free survival in patients with de novo DBLCL [153,154]. In addition, its levels in the serum of patients with ABC were higher than in those with GC-DBLCL and higher in disease stages III-IV compared to stages I-II [154]. Similarly, in a prospective cohort of 42 patients with classical HL, miR-21 plasmatic levels were associated with Hasenclever scores ≥3 and returned to normal at remission.

Using two independent quantitative approaches Fulci *et al.* [137] demonstrated an impressive over expression of miR-21 in leukemic CD19+ cells of B-CLL patients, with a 3.8 fold to a maximum of 10-fold increase compared to normal CD19 cells. Since the genomic locus encoding this miRNA was not amplified, they suggested that dysregulation of miR-21 occurred at a transcriptional or posttranscriptional level. This miRNA exhibits a significant anti apoptotic activity as already demonstrated in human glioblastoma cells in which it is highly expressed [155], and defective apoptosis plays an important pathogenic role in B-CLL. Accordingly, the over expression of miR-21 may be one of the initiating events in the leukemic transformation of B-cells [137]. In addition to promoting leukaemia initiation, miR-21 expression can stratify survival in patients with 17p deleted B-CLL as well as in cohorts of patients with B-CLL with a variety of chromosomal aberrations [156]. Contrary to that observed in DBLCL patients, high expression of miR-21 is a significantly unfavourable prognostic factor independent of other clinic-pathological factors in B-CLL patients. Recently, Medina *et al.* provided the first *in vivo* evidence that miR-21 can trigger B-lymphomagenesis, thus demonstrating that miR-21 is a robust oncogene *in vivo* [157]. Significantly, this landmark study not only verified that expression of miR-21 alone is sufficient to induce lymphomagenesis but also that direct shutdown of miR-21 can lead to lymphoma regression. Moreover, this first example of oncomir addiction highlights the central role that a single miRNA can have in the initiation, maintenance, and survival of oncomir-addicted cancers. The overexpression of miR-21 is likely to promote uncontrolled cell growth by interfering with a plethora of mRNAs encoding tumor suppressor proteins, and there is evidence that it mediates different downstream responses depending on the cell type and context. More specifically, *acidic nuclear phosphoprotein 32 family member A* (*ANP32A*), *Swi/Snf related, matrix associated, actin dependent regulator of chromatin A4* (*SMAR-CA4*) and *PTEN* are validated targets of miR-21 mainly in B-cell lymphoma [158], whereas *PTEN* and programmed cell death 4 (*PDCD4*) are involved in NK/T cell Lymphoma [159,160]. Particularly, given that both *PDCD4* and PI3K/PTEN/AKT inhibit physiologically tumor promotion and progression and intravasation [161,162], their strong repression suggests that miR-21 overexpression may contribute to the typical characteristics of aggressive clinical-pathological NK/T-cell lymphoma. Intriguingly, the removal of the miR-21 stimuli by a tetracycline antibiotic such as doxycycline or by miR-21 antisense oligonucleotides (ASO-21) [160] decreases the growth of NK/T-cell lymphoma lines via apoptosis. Since the PTEN-PI3K-AKT axis is also involved in the mechanisms of drug resistance, the use of antimiR-21 appears to be a promising therapeutic strategy in combination with standard agents to maximize the benefit for these high-risk patients.

### 6.2. miRNAs as Dysregulated Tumor Suppressor Genes in Lymphoid Malignancies

The miRNAs that physiologically up-regulate tumor suppressor genes and/or down-regulate oncogenes as well as interfere in cell survival and/or death may be regarded as tumor suppressor miRNAs since they usually prevent tumor development. Their decreased expression is related to tumor occurrence because it induces an abnormal cell growth as well as the loss of apoptosis function as a consequence of (1) the lack of negative control on the expression of oncogenes and/or genes that regulate cell differentiation and survival, and/or (2) the down-regulation of tumor suppressor genes. So far, a set of dysregulated tumor suppressor miRNAs such as miR-15a, miR-16-1, miR-181, miR-34a, miR-150, miR-30 and miR-29 has been identified in lymphomas.

The miR-15a/16-1 cluster is located at chromosome 13q14, a 30-kb region that is deleted in more than 50% of B-CLL patients [163] and whose genes are deleted or down-regulated in approximately 68% of B-CLLs [133]. Typically, down-regulation or loss of miR-15a/16-1 expression is associated with an indolent disease. The association between the frequency of the locus deletion and the recurrence of the same lymphoid histotype emphasizes the relationship between the miR-15a/16-1 cluster and the pathogenesis of B-CLL [133]. Interestingly, it has been reported that patients with monoallelic 13q14 deletion have slower growth kinetics than patients with biallelic deletion [164]. This underlines the existence of a relationship between the miR-15a/16-1 cluster and the pathogenesis of leukemia, and that residual expression of miR-15/miR-16 contributes to improve the prognosis of B-CLL patients. The anti-tumor effect of this cluster in B-CLL is due to the direct targeting of the anti-apoptotic protein Bcl2 [165] as well as *TP53*, *Cyclin Dependent Kinase 6* (*CDK6*) and *Myeloid cell leukemia sequence 1 (MCL1*), that is known as Bcl2-related [166]. Particularly, it may be assumed that a greater down-regulation of miR-15a/16-1 induces a lower suppression of Bcl2 anti-apoptotic activity, and consequently a greater tumor growth. *BCL2* is over expressed in the vast majority of patients [40,167] but only <5% of case shows mutations of the *BCL2* gene that can explain this aberrancy [168]. Accordingly it is also plausible that the miR-15a/16-1 cluster controls the functional translation of *BCL2* mRNA in many patients with B-CLL. The results of some studies [54,90,137,169] do not support however the concept of an inverse correlation between the status of 13q14 deletion and levels of miR-15a/16-1 expression in patients with B-CLL, as miR-15a/16-1 expression was indistinguishable from normal controls in patients bearing a hemizygous deletion. Furthermore, Fulci *et al.* [137] did not find significant increase of *BCL*2 expression levels despite the dramatic down-regulation of miR-15a/16-1, refusing the existence of an inverse correlation between miR-15a/16-1 and *BCL2* levels described by a previous study [165]. Accordingly, this finding refuted the hypothesis that in B-CLL the high levels of *BCL2* expression are secondary to a down-modulation of miR-15a/16-1. Similarly, Lawrie *et al.* [54] did not find any significant correlation between 13q14 deletion status and miR-15a/16-1 expression levels. On the other hand 13q14 deletion is detected in only about 60% of patients with B-CLL, so this abnormality cannot be primarily responsible for the disease and consequently the down-regulation/deletion of miR-15a/16-1 is just one of the potential pathogenic mechanisms responsible for the onset of B-CLL. The miR-15a/16-1 cluster modulates a myriad of oncogenes and tumor suppressor genes [76] that may potentially contribute to determining changes in the phenotype and aggressive behaviour of B-CLL. This issue has been addressed using transgenic mouse strains bearing conditional alleles that mimicked progressively more extended deletions from specific miR-15a/16-1 alone to (1) the entire B-CLL-associated minimal deleted region (MDR) of the 13q14 locus (*DLEU2* gene in addition to miR-15q/16-1 cluster) [170] or (2) the commonly deleted region (CDR) (genes *DLEU7* and *RNASEH2B*) [171]. In these experimental systems the phenotype of B-CLL correlated to the extent of the deleted region, since those mice with a MDR- or CDR-deleted region exhibited a more aggressive disease phenotype as compared with miR15-a/16-1 cluster deletion alone. This observation that other genetic components in addition to the miR-15a/16-1 cluster may contribute to the pathogenesis of B-CLL [171,172] does not preclude the role of miR-15a/16-1 but rather highlights the existence of other pathological mechanisms. Consistent with this view, it has been reported that a homologous cluster to miR-15a/16-1 (*i.e.*, miR-15a/16-2) is encoded at chromosome 3q25, a region that is not commonly deleted in B-CLL. This probably explains the residual miR-15a/16-1 activity in the more favourable prognosis of patients with 13q14 deletion as compared with those with other chromosomal deletions, such as 17p13 or 11q23. In accordance with this, Fabbri *et al.* [155] showed that miR-15a/16-1 cluster was linked in a molecular pathway with p53 that explained the pathogenic and prognostic implications (indolent *vs.* aggressive form) of recurrent 13q, 17p deletions in B-CLL. They demonstrated that in B-CLLs with 13q deletions the miR-15a/miR-16-1 cluster directly targeted *TP53* gene, whereby by a positive feedback loop the direct binding of *TP53* regulates the expression of miR-15a/16-1. Thus, the B-CLL pathogenesis and outcome was associated with this miRNA/*TP53* feedback circuitry, that provided a novel pathogenic model to explain the association of 13q deletions with the indolent form of B-CLL that involved microRNAs and *TP53* [166]. In view of the above findings, taken together with the description of rare B-CLL cases with 13q14 deletions where the deletion does not include the miR-15a/16-1 cluster [173]. It is likely that both the down-regulation of miR-15a/16-1 and 13q14 deletion are potential initial pathogenic mechanisms for the onset of B-CLL, but other pathways must be predominantly engaged to result in a full oncogenic activity in the disease. The down-regulation of miR-15a/16-1 is also thought to be a molecular event underlying the pathogenesis of other types of lymphomas such as MCL [174,175] and anaplastic lymphoma kinase (ALK)-positive anaplastic large cell lymphoma (ALCL) [176]. Specifically Zhang *et al.* [175] investigated the mechanisms of miR-15a/16-1 transcriptional repression and its epigenetic regulation by c-*MYC* and *histone deacetylase 3* (*HDAC*) in MCL, highlighting the existence of another important mechanism responsible for the down-regulation of miR-15a/16-1 expression in addition to 13q14 deletion. They noted that both c*-MYC* and *HDAC3* co-localized to the two promoters of the miR-15a/16-1 cluster gene, *DLEU2*, and that the inhibition of *HDAC* increased the histone acetylation of *DLEU2* promoters. This recruiting of *HDAC3* to down-regulate the expression of miR-15a/miR-16-1 was confirmed also by the luciferase reporter assay. Since the regulatory mechanism of miR-15a/16-1 was further demonstrated in BL, it is conceivable that the c*-MYC-*induced miR-15a/16-1 changes by *HDAC* may be a novel mechanism for *MYC*-driven miRNA suppression and malignant transformation in aggressive B-cell lymphoma [175]. Finally, Chen *et al.* [174] demonstrated that truncations of the Cyclin D1 (*CCND1*) mRNA, which impaired miR-16-1 binding sites, in MCL were associated with the pathogenesis of disease as a result of altered ability of *CCND1* to be down-regulated by this miRNA. The over expression of *CCND1*, a well-known proto-oncogene regulator of cell-cycle progression, was the hallmark of malignant transformation in MCL [177]. Furthermore it was shown that truncated *CCND1* mRNA correlated with poor prognosis in MCL [177]. Accordingly, the overexpression of *CCND1* due to its impaired down-regulation by miR-16-1 was another mechanism responsible to the pathogenesis of MCL in addition to the t (11;14) (q13;q32) translocation, the most well-known mechanism of overexpression found in MCL [174]. In the cases of MCLs with truncations of *CCND1*, the expression of miR-16-1 was regular whereas its regulation was aberrant. This confirms that the tumor development depends on the aberrant regulation by miRNAs, regardless of what generates this alteration. In ALK positive ALCL cases, Dejean *et al.* [176] demonstrated for the first time a strong inverse correlation between miR-16 and *VEGF* expression levels that depends on the ability of miR-16 to repress the translation of *VEGF* by directly interacting with its mRNA at the 3’-UTR region. Accordingly, ALK expression in concert with *hypoxia-induced factor 1a* (*HIF1a*) through the down-regulation of miR-16 increases both angiogenesis and tumor growth by up-regulating *VEGF* translation via the hypoxia miR-16 pathway. This study highlights another target of miRNA regulation, and consequently another pathogenic mechanism to tumor onset related to miRNA dysregulation.

MiR-181a and miR-181b are two members of the miR-181 family that act as tumor suppressor genes. They have been found differentially expressed in B-CLL [48]. The anti-tumor effect of miR-181b is mediated by directly targeting *T-cell leukemia/lymphoma 1* (*TCL1*), an oncogene that enhances the kinase activity of the oncoprotein AKT. AKT is critical for the transduction of antiapoptotic signals in B- and T-cells, and regulates several pathways involved in cell survival, cell proliferation and anti-apoptosis activity such as the mTOR, NF- kB, Mdm2 and CyclinD1 pathways [178]. By studying the mechanism of *TCL1* regulation in B-CLL, Pekarsky *et al.* [48] studied the regulation of *TCL1* expression by miR-181 and miR-29 in B-CLL patients in correlation of clinical outcome, observing three distinct miRNA signatures corresponding to three subgroups of B-CLL: indolent B-CLL, aggressive B-CLL and aggressive B-CLL showing 1q deletion. In particular, *TCL1* expression was correlated with disease aggressiveness as it was lower expressed in indolent disease. In normal B-cells, *TCL1* is regulated according to developmental stage with strong expression in resting B-cells, and loss of expression at the GC stage [179]. Several studies have demonstrated that this developmental pattern of regulation is largely correlated with the origin of disease resulting a strongly expression of *TCL1* in pre-GC tumor types (e.g., mantle cell lymphoma) and, conversely, a negative expression in post-GC subsets (e.g., multiple myeloma) [180]. The prognostic significance of Tcl1 levels has also been verified in transgenic mice models [181], and is further supported by the observation that high Tcl1 levels are associated with the presence of well-known “poor prognosis” markers such as ZAP-70 and unmutated IgVH status in B-CLL patients [179]. Similarly to the other members of the family, miR-181a directly targets the *BCL2* oncogene [45], thus providing further evidence of its anti-tumor effect in B-CLL. Indeed, considering the role of the miR-181 family in the early stage of B-cell differentiation and the biological disease features that are characterized by the progressive accumulation of mostly non-dividing CD5+ B-lymphocytes in the blood, bone marrow and lymphatic tissues, it is credible that the down-regulation of the miR-181 family by increasing the levels of two oncogenes, *TCL1* and *BCL2*, can lead to abnormal B-cell proliferation. Similarly, in the same study Pekarsky *et al.* found an inverse correlation between the expression of Tcl1 protein and miR-29b. Previously, it has been shown that the expression of miR-29 family members could discriminate between B-CLL samples with good and bad prognosis [90], suggesting that this finding may be related to the expression of *TCL1*. Santanam *et al.* [91] also studied the role of miR-29 in pathogenesis of B-CLL *in vivo* in transgenic mice overexpressing miR-29 in mouse B cells. This study suggested that dysregulation of miR-29 can contribute to the pathogenesis of indolent B-CLL. Finally, the role of miR-29a was also demonstrated in ALK-positive (ALK+) anaplastic large cell lymphomas (ALCL) which overexpress the major antiapoptotic protein *MCL-1.* Desjobert *et al.* [89] showed in ALK+ALCL cell lines and 20 biopsy specimens a low expression of miR-29a playing an important regulatory role in MCL-1 and that this down-modulation required an active NPM-ALK Kinase as in transgenic mice and *mouse embryonic fibroblast* (MEF) cells the absence of NPM-ALK resulted an increase of miR-29a expression. Furthermore, in ALCL cell lines and in a xenografted model the increased expression of miR-29a modulated apoptosis through inhibition of *MCL-1* expression, with a concomitant tumor growth reduction suggesting a potential new tool to affect tumorigenesis in these lymphomas.

MiR-34 is a pro-apoptotic and growth-suppressive miRNA whose expression is regulated positively by *TP53* [81,82,83,84,182] and, conversely, negatively by c-*MYC* [80]. In lymphomas, miR-34 is epigenetically silenced, mainly in NHL and in particular in NK/T-cell lymphoma where it is hypermethylated in a tumor-specific manner [183].

**Table 3:** Summary of miRNAs involved in lymphoma pathogenesis.

Ly Type	miRNA	Status	Func.	Target	BioM	Comment/Reference
**HL**	miR-135	DR		*JAK2*		The expression of miR-135a in cHL lymph nodes is down-regulated and correlates with clinical outcome. The miR-135a direct down-regulates the *JAK2*, thus affecting the expression of the antiapoptotic gene *BCL-XL*. In accordance with this the increased levels of miR-135a causes apoptosis and decreases cell growth [55]
	miR-155	UP	OG	*PU.1*	D	Specific biomarker of HL [43,71,72,143,144,145,146,147]
	let-7/miR-9	UP	OG	*PRDM1/blimp1*	D	[86,88]
	miR-17-92	UP	OG		D	Compared to other B-cell lymphoma cell lines, overexpression of the miR-17-92 cluster members miR-17-5p, miR-19a, miR-19b, miR-20a, and miR-92, is prominent in HL [43]
	miR-21	UP	OG	PTEN	D, P, PR	Plasmatic levels are associated with Hasenclever scores ≥ 3 and returned to normal at remission [42,43]. Involved in expression in cHL pathogenesis and is associated with therapeutic resistance [184]
	miR-150	DR	TSG		D	[43]
**DBLCL**	miR-155	UP in ABC	OG	*PU.1* *INPP5D (SHIP1)* *SMAD5*	D, P	More aggressive subtypes [140] Higher in ABC-DBLCL than in GC-DBLCL; useful for differential diagnosis of ABC-DBLCL and as a P considering the poor prognosis of ABC as compared with GC-DBLCL subtypes [71,72,138]. Evaluation of serum levels: 83% sensitivity, 65% specificity [139]. Inverse correlation between NF-kB/miR-155 and *PU.1*/CD10 expression [140]. Stimulation by TNF-α increases miR-155 expression that induces aberrantly activation of PI3K/AKT pathway, one the most important unfavourable P, by directly targeting *INPP5D* [66,67,137,140,141]. By targeting *SMAD5,* it makes DBLCL cells resistant to the growth-inhibitory effects of both TGF-beta1 and *BMP* [58,64,65,70,100]
	let-7f	UP	OG		D	[58]
	let-7b	UP in ABC	OG	*PRDM1/blimp1*	D	[88]
	miR-9	UP in ABC	OG	*PRDM1/blimp1*	D	[58,102]
	miR-15a	DR	TSG	*BCL2*	D	[2]
	miR-17-92 (miR-17-5b, miR- 9b)	UP in GC	OG	*BIM/PTEN*	P	Consistent with more aggressive phenotype [31,40,41,87] MiR-19b promotes cell proliferation and angiogenesis, represses apoptosis
	miR-21	UP in ABC	OG	*BCL2*	D, P	Elevated levels (in serum or biopsy) are associated with a better RFS in de novo DBLCL [138] More highly expressed in the poor risk ABC than in the GC-DBLCL subtype and higher in disease stages III-IV compared to stages I-II [138,153,154]. Conversely in B-CLL [46,156,158]
	miR-30	DR	TSG	*BCL6* *PRDM1*		[50,85]
	miR-34a	DR	OG			
	miR-150	DR	TSG	*c-Myb*		[18,41]
**MCL**	miR-17-92	UP	OG	*E2F1* *(c-MYC)* *PHLPP2* *PTEN* *BIM*	D, PR	Enhances resistance to chemo- [37] and radiotherapy via PI3K/AKT by targeting PTEN and PHLPP2 [152] [27,31,34,35,37,152]
	miR-16-1	1. BS-del 2. DR	TSG	*CCDN1*	P	1. Truncation in *CCDN1* mRNA alters its ability to be down-regulated by miR-16-1, resulting in MCL development and correlating with poor prognosis [174] 2. Myc represses miR-15/16-1 expression through recruitment of *HDAC3* [175].
	miR-181c	UP				[57,58]
	miR-155	UP	OG			[185]
**FL**	miR-9	UP		*PRDM1/blimp1*	D	MiR-9 (-5p, -3p) is significantly upregulated; Activated by MYC. It regulates *NF-kB* and down-regulates *PRMD1/BLIMP1* [41,58]
	let-7	UP	TSG	*PRDM1/blimp1*	D	Reduced *PRDM1* levels are important in FL characterized by the tightly regulated expression of *BCL6* and *PRDM1* [86,87].
	miR-155	UP				[41]
**BL**	let-7a, let-7c, let-7e, let-7-f	DR		*PRDM1/blimp1*		Loss of the let-7 (a; c) participates to the genesis and maintenance of the lymphoma phenotype through *c-MYC* regulation [186]
	miR-9	UP	OG			
	miR-17-92	UP				miR-17-3p, miR-18a, miR-19a, miR-19b, miR-92 up-regulated in BL *vs.* NHL [58]
	miR-29	DR/lost	TSG	*TP53* *TCL1*	D	MiR-29 family regulates *TP53* [27,58] MiR-29b, regulates *TCL-1* expression, whereby the aberrantly expression of *TCL1* in BL has been proposed as a diagnostic marker [48,58] MiR-29 is negatively correlated with *MCL-1*.
	miR-34b	DR				Targeted by *TP53*
	miR-150	DR	TSG			[18,58]
	miR-155	DR/lost				MiR-155 is the most significantly lost miRNA in BL [72] followed by miR-29b and miR-146a [58], this making it one of the most suitable markers for differential diagnosis between BL *vs.* DBLCL [44,75].
	miR-15a/miR-16-1	DR/del.	TSG	*BCL2* *TP53*	D, P	Deleted (region 13q) or down-regulated in ≈ 68% of B-CLLs [133].
	miR-15a/miR-16-1	DR/del.	TSG	*BCL2* *TP53*	D, P	It is associated with pathogenesis and outcome of B-CLLs: monoallelic deletion slower growth kinetic than biallelic [164] due to the directly targeting of *TP53* [166] and consistent with indolent B-CLL and more favourable prognosis than 17p13 or 11q23 deletions [90]. In addition targets *BCL2, MCL1 and CDK6* [165]
	miR-17-92	UP	OG		D	Specifically: miR-19a, miR-20a, miR-92 [43]
**BL**	miR-21	UP	OG	*ANP32A* *SMAR-CA4* *PTEN*	P	Dramatically overexpression without genomic loci amplification: dysregulation at post- or transcriptional level [137] High expression is a significantly unfavourable P independent of other clinic-pathological factors in B-CLL patients [156] Can trigger B-lymphomagenesis by targeting *ANP32A*, *SMAR-CA4* and *PTEN* [157,158]
	miR-29	DR	TSG	*TCL1*	P	Expression of members of miR-29 family could discriminate between good and bad prognosis CLL samples as results of *TCL1* targeting. MiR-29b is down-regulated in aggressive and poor prognosis B-CLLs which are characterized by high levels of *TCL1* [48,90]. MiR-29c is associated with TSF and OS [187].
	miR-34	DR			P	Aggressive B-CLL: in 11q deleted CLL with high levels of ZAP-70 [166].
	miR-181b	DR	TSG	*TCL1*	P	Aggressive B-CLL. DR in poor prognosis.Down-regulation of *TCL1* results the activation mTOR, NF- kB, Mdm2 and CyclinD1 pathways [178]. So its expression correlates with disease aggressiveness: high expression in aggressive B-CLL, and lower in indolent disease [48]
	miR-181a	DR	TSG	*BCL2*		[45]
	miR-150	UP	TSG	*c-MYB*		[18,58]
	miR-155	UP	OG		P	Aggressive B-CLL. Increased expression for dysregulation at post- or transcriptional level. [71,90,137]
**MZL**	miR-9	UP	OG	*NF-κB*		[41,44]
	miR-155	UP	OG			[41,71]
	miR-200a, miR-200b, miR-200c	UP		*ZEB1* *ZEB2*		miR-200 family inhibits the initiating step of metastasis, the EMT, by maintaining the epithelial phenotype through directly targeting the transcriptional repressors of E-cadherin, *ZEB1* and *ZEB2* [58]
	miR-126	DR				[58]
**NK/T Ly**	miR-21	UP	OG	*PDCD4* *PTEN*		Overexpression may contribute to the typical aggressiveness of NK/T cell lymphoma by the strong repression of *PDCD4* and PI3K/PTEN/AKT [159,160,161,162]
	miR-34a	DR	TSG			Hypermethylated in a tumor specific manner [55]
	miR-150	DR	TSG	*DKC1* *AKT2*		Potential causative event for the onset and progression of NK/T cell lymphoma since the transduction of miR-150 decreases cell proliferation and induces apoptosis into NK/T cell lymphoma lines [56]
**ALCL**	miR-29a		TSG			miR-29a targets *MCL-1* that could promote tumor cell survival by inhibiting apoptosis. This down-modulation requires an active NPM-ALK Kinase since the absence of kinase results an increase of miR-29a expression. In ALCL cell lines and in a xenografted model the increased expression of miR-29a modulated apoptosis through inhibition of *MCL-1* expression, with a concomitant tumor growth reduction suggesting a potential new tool to affect tumorigenesis in these lymphomas [89].
**ALCL**	miR-16			*VEGF* *HIF-1a*		Down-regulation of miR-16 increases both angiogenesis and tumor growth by up-regulating VEGF translation via the hypoxia-miR-16 pathway [176]
	let-7	UP	OG	*PRDM1/blimp1*		[56]
**B-NHLs**	miR-15a/16-1	DR/	TSG			*c-MYC*-induced miR-15a/16-1 repression by HDAC may be a mechanism for malignant transformation in aggressive B-cell lymphoma [175]. Deletion in indolent disease.
	miR-21	UP	OG	*ANP32A* *SMAR-CA4* *PTEN*	D, P	Key role in B lymphomagenesis [157,158]
	miR-17-92	UP	OG	*BIM* *PTEN* *E2F1* *PHLPP2*	D, P, PR	Located in 13q31-q32 region frequently (65%) amplified in lymphomas, especially in aggressive B-NHLs.Oncogenic effects depend on the Bim, *PTEN* and *E2F1* targeting [30,34].
	miR-34a	UP	OG	*SIRT1*		Confers drug resistance in B cells that overexpress *MYC* [79,81]
	miR-155	UP	OG			Distinguishes ABC-DLBCL from GC-DLBCL [71,138]. In more aggressive B-NHLs aberrant activation of NF-kB increases expression of miR-155, which then down-regulates PU.1, and consequently leads to reduced CD10 expression [140]

Ly type, Lymphoma type; Func. function associated to miRNA; BioM., Bio Marker; CLL, Chronic lymphocytic leukemia; DLBCL, Diffuse large B-cell lymphoma; HL, Hodgkin lymphoma; MCL, Mantle cell lymphoma; MZL, Marginal zone lymphoma; BL, Burkitt’s lymphoma; FL, Follicular lymphoma; NK/T Ly, Natural Killer/T lymphoma; ALCL, anaplastic large-cell lymphomas. D = Diagnostic Biomarker; P = prognostic Biomarker; PR = Predictive of response to treatment Biomarker; OS = Overall survival; RFS = Relapse free survival; TFS = treatment-free survival; EMT= epithelial-mesenchymal transition; BS-del = Binding site deleted; UP = Up regulated; DR= Down regulated.

MiR-150 is a tumor suppressor gene that regulates B-cell development [18] and NK T cell function by targeting *MYB* [55,56], as well as controlling the other T-cell subsets interacting with the *NOTCH3* signal [120]. The deregulation of miR-150 can be a causative event for the onset and progression of NK/T cell lymphoma since the transduction of miR-150 decreases cell proliferation and induces apoptosis into NK/T cell lymphoma lines [56]. However, it should be noted that several miRNAs could converge and cooperate to regulate/alter the signal pathways of NK/T-cell malignancy. For example, as previously mentioned the over-expression of miR-21 and miR-155 also leads to tumor growth suppression [160]. It has been suggested that the anti-tumor effects of miR-150 are related to the decrease of cell proliferation associated with increased cellular senescence with low telomerase activity as well as the increase of apoptosis. Molecularly, miR-150 directly targets *Dyskerin 1* (*DKC1*) which is a critical regulator of telomerase activity, and deregulates *AKT2*, thus increasing the levels of two pro-apoptotic tumor suppressors, *BIM* and *TP53* [56]. Finally, miR-30 is described as a marker of the germinal centre involved in the pathogenesis of DBLCL [85] where it exerts its anti-tumor activity by targeting *BCL6* and *PRDM1* [50].

The miR-135 is another tumor suppressor gene whose down-regulation is involved in lymphoma tumorigenesis. In lymph nodes of cHL patients the expression of miR-135a correlates with clinical outcome. Navarro *et al.* [55] examined the influence of 25 miRNA signatures for cHL on clinical outcome in 89 homogeneously treated cHL patients with a median follow-up of 80 months. They observed that patients with low miR-135a expression had a higher probability of relapse and a shorter disease-free survival. Furthermore, the increased levels of miR-135a after pre miR-135a transfection in cHL cells lines caused apoptosis and decreased cell growth. By functional analysis of cHL cell lines they showed that the miR-135a directly targeted the Janus Kinase *JAK2.* The down-regulation of *JAK2* led to decreased mRNA and protein levels of the antiapoptotic gene *BCL-XL*, suggesting a role for *BCL-XL* in miR-135a/JAK2–mediated apoptosis. This was in accordance with the finding that miR-135a play a critical role in the survival of cHL cells and the prognosis of cHL patients, suggesting a new therapeutic approach of cHL patients through the targeting miR-135a [55].

Finally, miR-9 [86,88] and let-7 [88] are up-regulated in HL cell lines, although the mechanisms responsible for their high levels remain to be elucidated. Their overexpression implicates down-regulation of *PRDM1/BLIMP1*, a master regulator for B-cell terminal differentiation that acts as a tumor suppressor gene also in non-GC-DBLCL, particularly in ABC-DBLCL, and anaplastic large T-cell lymphoma [56,102] Indeed, Nie *et al.* [86] demonstrated that *PRDM1/blimp1* was down regulated by miR-9 and let-7a in Hodgkin/Reed-Sternberg (HRS) cells of HL. Specifically, miRNA expression profiling by direct miRNA cloning demonstrated that both of these miRNAs are among the most highly expressed in cultured HRS cells and when over-expressed in HL cell lines correlated with low levels of *PRDM1/blimp1*. Similarly, the majority of HRS cells in primary HL cases showed weak or absence of *PRDM1/blimp1* expression [86]. Previously data demonstrated that *PRDM1* played a key role in the pathogenesis of human lymphomas being inactivated by a classic mechanism for tumor suppressor genes in non-GCB-DLBCL [102,103]. Pasqualucci *et al.* [102] reported that *PRDM1/blimp1* gene was inactivated by structural alteration in 24% ABC-DBLCL, but not in GC-DBLCL or unclassified DLBCL. Structural alterations including gene truncations, nonsense mutations, frameshift deletions, and splice site mutations generated aberrant transcripts encoding truncated blimp1 proteins. However, most non–GC type DLBCL cases (77%) lacked blimp1 protein expression, despite the presence of blimp1 mRNA, suggesting that the same gene was inactivated by epigenetic mechanisms in an additional large number of cases. These findings indicate a role as a tumor suppressor gene for *PRDM1/blimp1*, whose inactivation may contribute to lymphomagenesis by blocking post-GC differentiation of B cells toward plasma cells. In addition, *BCL-6*-mediated transcription repression of *PRDM1* caused blockade of terminal differentiation in GC-type DLBCL. In line with this, Nie *et al.* [88] demonstrated the presence of alternative mechanisms of down-regulating *PRDM1* in a subset of DLBCL characterized for without *PRDM1* mutations, consistent with translational down-regulation by miRNA let-7 family. This subset, exhibiting relatively high *PRDM1* mRNA levels but low levels of PRDM1 protein, showed over-expression of let-7 (in particular let-7b) as compared with normal GCB cells, suggesting an abnormal epigenetic down-regulation of *PRDM1* by let-7. This may represent an alternative mechanism of reducing normal PRDM1 function in DBLCL cases without *PRDM1* mutations.

### 6.3. miRNAs as Diagnostic or Prognostic Tool in Lymphoid Malignancies

Some individual miRNAs are differently expressed in lymphoma histological subtypes. For example miR-155 is highly expressed in the main B-NHL subtypes including DBLCL, MCL and HL, but is down-regulated in adult BL. The assessment of miR-155 expression can therefore be especially useful in borderline cases in which its different expression can help to distinguish between two entities such as DBLCL and BL. However, most studies propose a combination of miRNAs rather than a single miRNA for discriminating lymphoma subtypes. In fact, unsupervised hierarchical clustering analysis revealed specific miRNA signatures associated with many distinct lymphoid malignancies. The most cited example of a unique miRNA expression profile being associated with a specific lymphoma is observed in B-CLL cases with 13q13.4 deletion [43,54,76,90,137,169,170,171,172,187]. As previously mentioned miR-15a and miR-16-1 are located in the smallest region of the deletion at 13q13.4 and are frequently deleted or down-regulated in B-CLL cells [133]. This implies a significant difference in miRNome expression between B-CLL samples and normal CD5+ B-cells, which suggests the possibility to use a miRNA profile to distinguish normal B-cells from malignant B-cells [76]. Furthermore, miRNA expression patterns can have relevance to the biological and clinical behaviour of this leukemia. In fact, Calin *et al.* [90] identified a unique miRNA expression signature associated with prognostic factors and disease progression in B-CLL. They investigated whether a specific miRNA signature was associated with four subgroups of 94 B-CLL patients with different clinical courses: group 1 (expression of ZAP-70 and unmutated IgVH), group 2 (expression of ZAP-70 and mutated IgVH ), group 3 (no expression of ZAP-70 and unmutated IgVH ), and group 4 (no expression of ZAP-70 and mutated IgVH ).

Out of 13 miRNAs investigated they found 9 (miR-15a, miR-195, miR-221, miR-23b, miR-155, miR-24-1, miR-146, miR-16-1, miR-16-2) significantly over expressed in group 1, the group with poor prognosis. This miRNA signature profile may be relevant for the pathogenesis of B-CLL because it consists of miRNAs that are abnormally expressed in B-CLL (miR-15a and miR-16-1) or other leukemias (miR-155) or are located in regions involved in cancers (miR-23b, miR-24-1, miR-29b-2, and miR-195) [130,131,188]. Importantly, the study of Calin *et al.* demonstrated that miRNA signature was associated with ZAP-70 expression and IgVH mutation status, two factors that are predictive of disease progression and poor outcome [90]. Using prediction analysis for microarrays (PAM) and survival analysis they also observed a significant relationship between the expression of 9 miRNAs and the time from diagnosis to beginning of chemotherapy. This is another important factor associated with disease aggressiveness since patients are not usually treated until the disease is advanced or symptoms develop [189]. These data are the most classic example of the relationship between a specific miRNA signature and lymphoma prognosis and progression. Other specific miRNA signatures are associated with the histopathological and clinical features of lymphomas, and thus miRNAs are emerging as powerful diagnostic, prognostic and predictive markers. For example, using qRT-PCR to assess the expression of 157 miRNAs in 49 lymph nodes from cHL and 10 reactive lymph nodes (RLNs), Navarro *et al.* [42] identified a specific miRNA signature in three well-defined subgroups: nodular sclerosis cHL, mixed cellularity cHL and RLNs. In detail, a distinctive signature of 25 miRNAs discriminated lymphoma from the control cases, and 36 differentially expressed miRNAs distinguished the mixed cellularity subtype of cHL from nodular sclerosis. MiR-96, miR-128a, and miR-128b were selectively down-regulated in cHL with EBV [42]. Recent data concerning the prognostic value of miRNA profile in cHL showed that miR-21, miR-30e, miR-30d and miR-92b were able to identify two different risk groups for 5-year failure-free survival (FFS) among 168 patients [184]. Similarly Roehle *et al.* [41] analysed the expression signatures of 157 miRNAs in 58 DLBCL, 46 FL and 7 non-neoplastic lymph nodes (LN). They found specific DLBCL- and FL- signatures, which comprised miRNAs involved in haematopoiesis (miR-150 and miR-155) or tumour development (miR-210, miR-10a, miR-17-5p and miR-145). Specifically a lymphoma-specific aberrant expression of miR-9/9*, miR-301, miR-338 and miR-213 was observed in FL, whereas miR-150, miR-17-5p, miR-145 and miR-328 constituted a specific miRNA signature for DLBCL. In addition, this group demonstrated that mir-330, mir-17-5p, mir-106a and mir-210 correctly classified 98% of DLBCL, FL and non-diseased samples. Finally, eight miRNAs, including miR-21, miR-127, miR-34a, miR-195 and let7g were found to correlate with event-free and overall survival in DBLCL [41]. Lawrie *et al.* [87,138] investigated if transformation of FL in more aggressive DLBCL was associated with changes in miRNA expression, comparing (a) transformed DLBCL with de novo DLBCL, and (b) FL that underwent subsequent transformation with FL that had no transformation at a median follow-up of 60 months. They first showed that ABC and GC subtypes of DLBCL have a distinct miRNA expression profile as miR-155, miR-21 and miR-221 were more highly expressed in ABC-type than GC-type cells, allowing to distinguish between ABC- and GC-DBLCL cases. [138]. Notably, 14 differentially expressed miRNAs were able to correctly predict >85% of transformed *versus* de novo DLBCL cases, and a signature of 6 miRNAs (miR-223, miR-217, miR-222, miR-221, let-7i and let-7b) accurately predicted transformation of FL. Specifically an increased level of miR-17-92 cluster expression was consistent with a more aggressive clinical phenotype of DLBCL [87]. These findings suggest that miRNAs play a potential role as diagnostic and prognostic markers in these lymphomas; they may be used to identify FL patients at risk of high-grade transformation, and the same conclusions could be reached about the other subtypes of lymphomas.

Recent studies have highlighted another aspect of miRNA application concerning the crucial role of these molecules in drug resistance development, or conversely in the enhancement of sensibility to a specific chemo-treatment. For instance, Alencar *et al.* [190] demonstrated that miR-181a, miR-22 and miR-18a were independent prognostic indicators of survival in R-CHOP (rituximab, cyclophosphamide, doxorubicin, vincristine, prednisolone) treated DLBCL patients. In another study, as previously mentioned Rao *et al.* [37] demonstrated that the overexpression of miRNA-17-92 cluster in MCL by directly targeting *PHLPP2*, in addition to *PTEN* and *BIM*, inhibited chemotherapy-induced apoptosis and enhanced tumor growth via PI3K/AKT pathway activation, as *PHLPP2* was a negative regulator of this pathway. Furthermore, they confirmed their results by demonstrating that in a xenograft MCL mouse model the inhibition of miR-17-92 expression suppressed the PI3K/AKT pathway and inhibited tumor growth. Given the insufficient efficacy of standard treatments in poor risk patients with MCL, novel therapeutic approaches that target the miR-17-92 cluster appear an attractive option for MCL patients [37]. Similarly, Jiang *et al.* [152] demonstrated that the over expression of miR-17-92 significantly increased the radio-resistance of human MCL cells via PI3K/AKT pathway by targeting *PTEN* and *PHLPP2*. They showed that the overexpression of this miRNA significantly increased survival cell number, cell proliferation and decreased cell death of human MCL cells Z138c after different doses of radiation. This was due to down-modulation of *PTEN* and *PHLPP2*, and enhancing of Akt activity after over-expressing miRNA-17-92 after irradiation. This suggested miR-17-92 as novel target molecule to enhancing radiotherapy sensitivity of MCL in clinic [152] (Table 4).

**Table 4:** Some specific miRNA signatures lymphoma-associated or treatment response correlated.

Lymphomas	Signatures	St.	Comments/Ref.
**DBLCL**	miR-210, miR-155, miR-106a, miR-17-5p	UP	Significantly higher expression in DBLCL than RLN [41]
	miR-150, miR-145, miR-328, miR-139, miR-95, miR-99a, miR-10a, miR-149, miR-320, miR-151, let-7e (miR-17-3, miR-595, miR-663)	DR/lost	Significantly lower expression in DBLCL than RLN [41] miR-17-3, miR-595, miR-663 most significantly lost in DBLCL [58]
**FL**	miR-9, miR-301, miR-213, miR-9*, miR-330, miR-106a, miR-338, miR-155, miR-210	UP	Significantly higher expression in FL than RLN [41]
	miR-320, miR-149, miR-139	DR	Significantly lower expression in FL than RLN [41]
**DBLCL *vs.* FL**	miR-150, miR-17-5p, miR-145, miR-328 *vs.* miR-9/9*, miR-301, miR-338 and miR-213		Differentially expressed miRNAs in DBLCL and FL identify signatures respectively in DBLCL and FL [41]
**DBLCL/FL/RNL**	mir-330, mir-17-5p, mir-106a and mir-210		Correctly identifies 98% of DLBCL, FL and RLN [41]
**ABC-DBLCL** ***vs.*** **GC-DBLCL**	miR-155, miR-21 and miR-221	UP	More highly expressed in ABC- than GC-subtypes, distinguish between ABC- and GC-DBLCL cases (*p* < 0.05). MiR-21 expression is an independent prognostic indicator in de novo DLBCL (*p* < 0.05) [138]
**DBLCL-dn** ***vs.*** **DBLCL-t**	miR-27a, miR-19b, miR-25, miR-18a, miR-636, miR-92, miR-621, miR-526c, miR-766, miR-299-5p, miR-380-3p, miR-129, miR-588	UP	More highly expressed (*p* < 0.05) in DLBCL *de novo* than DLBCL-t cases, correctly predict transformation >85% [87]
**FL-t *vs.* FL-nt**	miR-223, miR-217, miR-222, miR-221, let-7i, miR-7b	UP	Differentially expressed (*p* < 0.05) between FL-t and FL-nt, accurately (89%) predict transformation of FL cases [87]
**DBLCL prognosis**	miR-637, miR-608 and miR-302		Poor prognosis [87]
	miR-330, miR-30e, miR-425, miR-27a, miR-24, miR-23a, miR-199b, miR-199a* and miR-100		Better outcome [87]
	miR-21, miR-127, miR-34a, miR-195, let-7g, miR-19a, miR-27a		Correlate with EFS and OS [41]
**DBLCL drug sensitivity**	miR-181a, miR-22 and miR-18a		Independent prognostic indicators of survival in R-CHOP treated DBLCL [190]
**B-CLL poor prognosis**	miR-29		
	miR-15a, miR-195, miR-221, miR-23b, miR-155, miR-24-1, miR-146, miR-16-1, miR-16-2	UP	Significant relationship between the expression of 9 miRNAs and the time from diagnosis to beginning of chemotherapy [90]
**CLL *vs.* B-NHL**	miR-182, miR-199a*(5p), let-7 family, miR-424, miR-10a, miR-7, mir-126, miR-218, MiR-197, miR-595 , miR-483	DR/UP	MiR-197 the most highly expressed miRNA, miR-595 and miR-483 also upregulated [58]
**BL *vs.* B-NHL**	miR-17-3p, miR-18a, miR-19a, miR-19b, miR-92	UP	Up/Down-regulated in BL *vs.* NHL [58]
	let-7 family, miR-29 (a, b, c), miR-155, miR-146a	DR	
**HL**	miR-17-92 cluster members, miR-16, miR-21, miR-24, and miR-155.miR-150	UP DR	The HL-specific miRNAs up-regulated [43]. Only miR-150 is significantly down-regulated in HL compared with NHL [43].
**cHL *vs.* HL EBV+**	miR-96, miR-128a, miR-128b	DR	Selectively down-regulated in HL lymph nodes of EBV+ HL patients [42]
**cHL prognosis**	miR-135a		Expression of miR-135a in HL lymph nodes correlates with clinical outcome. Patients with low miR-135a expression had a higher probability of relapse and a shorter disease- free survival. [55]
	miR-21, miR-30e, miR-30d and miR-92b		To identify two different risk groups for 5-year FFS [184].

St., Status of expression; CLL, chronic lymphocytic leukemia; DLBCL, Diffuse large B-cell lymphoma; cHL, classical Hodgkin lymphoma; BL, Burkitt’s lymphoma; FL, Follicular lymphoma; DLBCL-dn, DBLCL de novo; ABC-, Activated B Cells; GC, Germinal Center; DBLCL-t, DBLCL transformed; FL-nt, Follicular lymphoma not transforming; FL-t, Follicular lymphoma transforming; RLN, Reactive lymph nodes; D = Diagnostic Biomarker; P = prognostic Biomarker; PR = Predictive of response to treatment Biomarker; OS = Overall survival; EFS = Event free survival; DR = Down-regulated; UP, Up-regulated.

Finally, there is a huge growth of interest in the area of circulating miRNAs, which could be non-invasive biomarkers for lymphoma patients. Besides their aforementioned use as biomarkers of circulating miR-155 and miR-21 in B-lymphomas, plasma miR-21, miR-494 and miR-1973 have distinct kinetics during therapy in cHL and have the potential to greatly assist clinical decision-making and aid interpretation of PET/CT [191]. Notably, functional analyses suggested that miR-21 is involved in expression in cHL pathogenesis and is associated with therapeutic resistance [184].

## 7. Novel Therapeutic Strategies by Modulation of miRNAs

As briefly discussed above, increasing evidence implicates miRNAs as key regulators of critical signaling cascades that govern cell fate and the function of normal and pathological lymphoid progenitor/precursor cells. Moreover, the presence of recurrent miRNA expression profiles, which mark specific lymphomas, highlights the contributory role of miRNAs in lymphoma pathogenesis. These discoveries not only provide important new tools to refine and deliver greater accuracy in diagnosis, prognostication and treatment planning/stratification at the bedside [123,192] but also offer clinicians exciting prospects for novel therapeutic strategies. Although this field of research is in its infancy with a relative paucity of data and many unresolved issues, *in vitro* and *in vivo* preclinical studies suggest that the supplementation or inhibition of specific miRNAs can alter essential signaling of malignant cell survival or overcome mechanisms of drug resistance. These promises attract special attention from both academia and biotechnology companies on the modulation of specific miRNAs as a useful option alone or in combination with other therapies for the treatment of cancer patients. To this purpose, several aforementioned studies in transgenic murine models have been carried out to assess the functional role of miRNAs and their involvement in tumor development and to explore the effects of modulation of miRNA expression. These studies demonstrated that pharmacological interventions designed to correct abnormal miRNA expression and/or injection of miRNA mimics can suppress cancer formation and progression, and the first cancer-targeted miRNA drug MRX34, a liposomal formulation loaded with tumor suppressor miR-34a mimic, entered a Phase I clinical trial in patients with primary liver cancer or those with liver metastasis from other cancers in April 2013 [193].

Depending on the function of miRNAs and their aberrant status in malignancies, they are generally classified as oncomirs or tumor suppressor genes. These give rise respectively to the following two major therapeutic approaches:

1. Inhibition of tumor-inducing miRNAs: *miRNAs as therapeutic targets*;
2. MiRNA replacement by re-introducing miRNAs with tumor suppressor functions: *miRNAs as therapeutic agents*.

The first approach is based on the use of antisense single-stranded oligonucleotides complementary to miRNAs, functioning as anti-miRNAs to silence or block a target miRNA that acts as an oncogene. Chemically modified anti-miRNAs, such as cholesterol-conjugated “antagomirs”, locked nucleic acid oligonucleotides, and polylysine-conjugated peptide nucleic acids (PNAs) have demonstrated effectiveness *in vivo* [194,195,196]. This therapeutic approach can be applied to specific neoplasms in which the overexpression of a particular miRNA is the hallmark of cell proliferation and is causally involved in disease onset and/or progression (such as miR-155, miR-17-92 cluster, miR-9 and let7 which are overexpressed in many lymphomas).

Chemically modified anti-miRNAs can also be used to reverse drug resistance induced by dysregulation of a given miRNA. For example, as previously mentioned, it has been shown that miR-17-92 cluster overexpression significantly enhances resistance to radiotherapy in human MCL, and therefore it may be a potential molecular target for improving the effectiveness of conventional treatments. Further evidence of this premise comes also from the fact that antagomir 17-5p abolishes *in vivo* the growth of therapy-resistant neuroblastoma cells which express the miR-17-92 cluster at an elevated level [80,197]. Overall, strategies using miRNAs as therapeutic targets in lymphoma may be directed, in principle, to dampening the oncogene/miRNA pathway or to silencing miRNAs, inducing chemoresistance.

So far, in many studies both *in vivo* and *in vitro*, several miRNAs have been silenced by antisense oligonucleotides such as miR-155, miR-17-92, and miR-21 but the possibility to use anti-miRNAs as drugs in clinical practice is still a long way from being realized since a number of obstacles still have to be overcome. These include the achievement of stability, safety and successful delivery of therapeutic anti-miRNAs to the appropriate tissue and into the cells [198]. Nevertheless, there are some anti-miRNAs in therapeutic development, including the first published phase II study carried out in 36 patients with chronic HCV genotype 1 infection. This study evaluated the safety and efficacy of Miravirsen, a locked nucleic acid–modified DNA phosphorothioate antisense oligonucleotide that sequesters mature miR-122 [199]. Since the release of therapeutic anti-miRNAs to the appropriate tissue is not influenced by chemical modifications, delivery can be engineered using viral and non-viral carriers, aiming at reaching tumor-specific systems. In this regard, Babar *et al.* [200] recently developed one of the most appealing technologies. They described a sophisticated technology based on nanoparticles coated with a cell-penetrating peptide encapsulating antisense peptide nucleic acids to systemically deliver anti-miR-155 (antagomir) *in vivo* in a B-lymphoma mouse model. In this study, overexpression of miR-155 was induced in the lymphoid tissues of a deficient miR-155 mouse model, causing disseminated lymphoma characterized by clonal transplantable pre B-cells. In this inducible system, nanoparticle-based therapy targeting miR-155 resulted able to reduce lymphoma growth. In particular, nanoparticles injected *in situ* or systemically produced a rapid regression of lymphadenopathy, partially due to apoptosis of the malignant B-lymphocytes.

The second promising strategy to therapeutically modulate miRNAs consists in the reintroduction or re-establishment of physiological levels of down-regulated miRNAs with tumor-suppressive function. Mostly, this result could be achieved through exogenous introduction into diseased tissues or systemic administration of short double-stranded miRNA mimics, functioning similarly to endogenous miRNAs. A miRNA for which the tumor suppressor role is well-defined is miR-34. Since the miR-34 gene is located in regions that have been associated with fragile sites of the genome that are frequently altered, a loss of miR-34a expression has been found in a wide range of malignancies, including lymphomas and leukemia. Therefore, as mentioned above, miR-34a mimic formulations are promising drugs now in development for clinical use in solid tumors. In the lymphoma field, using a xenograft DBLCL model Craig *et al.* [201] explored the *in vivo* relevance of miR-34 dysregulation in lymphomagenesis by inoculating an ABC-type cell line (U2932) expressing minimal endogenous levels of miR-34a in humanized immunodeficient NOD/SCID/IL2RG^−/−^ mice. In this model intravenous injections of a neutral lipid emulsion (NLE)/miR-34a formulation in mice with established subcutaneous U2932 lymphoma reduced tumor growth by 76%, due to apoptosis of the malignant B-lymphocytes [201]. Notably, other functions of miR-34a can be seen in the case of chemoresistance. MiR-34a supplementation confers chemosensitivity mainly through modulation of p53, and it produces a synergic effect with cytotoxic chemotherapeutic agents [202] as well as target therapies [203] in many cancers and haematological malignancies. Intriguing new data suggest that the tumor suppressor effects of a given miRNA could also be restored by removing its epigenetic silencing with epigenetic drugs like DNA-demethylating agents that change the DNA methylation status (e.g., 5-aza-2′-deoxycytidine and zeburaline) or histone deacetylase inhibitors (e.g., trichostatin A). Support for this therapeutic option comes from genetic studies suggesting that hypermethylation of their corresponding promoter loci is another mechanism that can lead to reduced levels of endogenous miRNAs with tumor suppressor activity. On the other hand the epigenetic down-regulation of miRNA expression by hypermethylation is involved in tumorigenesis or represents an adverse prognostic factor associated with poor disease-free and overall survival [204]. In this regard, it is worth mentioning that among haematological malignancies, miR-34a is preferentially hypermethylated in NHL, and particularly in NK/T-cell lymphoma, in a tumor-specific manner. As well as targeting miR-34 in lymphoma, pharmacological reversal of epigenetic silencing by inhibitors can be employed to re-express other miRNAs. For example, in acute lymphoblastic leukemia (ALL) miR-124 is epigenetically down-regulated, and this confers a poor prognosis in ALL patients due to a high relapse and mortality rate [205]. In addition, Saito *et al.* [206] demonstrated that chromatin-modifying drugs (5-aza-20-deoxycytidine) and/or the *HDAC* inhibitor 4-phenylbutyric acid (PBA) induce the specific activation of miR-127 along with the down-regulation of its target, the proto-oncogene *BCL6* in human cancer cell lines, including Ramos lymphoma cell line. Thus the reactivation of miR-127 by histone modification can be a further potentially successful option for cancer treatment. This is due to the consequent repression of the proto-oncogene *BCL6* expression [206] whose dysregulation in part directly or indirectly represses *PRDM1* and is implicated in the pathogenesis of B-cell lymphoma, mainly of DLBCL [207].

As previously discussed, emerging evidence indicates that miRNAs are also determinants of drug sensitivity/resistance [208]. Consequently another very reliable therapeutic opportunity is the combination of miRNA modulating agents with anticancer drugs in order to improve the effectiveness of conventional treatment. Even though this issue was successfully addressed in preclinical models, no combination has been explored yet in clinical trials because the underlying molecular mechanisms are very complex and not fully understood which could lead to unwanted results. For instance, Sotillo *et al.* [79] showed surprisingly that the restoration of miR-34a levels in B lymphoid cells along with *MYC* overexpression confers drug resistance because miR-34 inhibits the *TP53*-dependent apoptosis induced by bortezomib. This finding appears in contrast to the assumption that miR-34a acts as a tumor suppressor gene by improving the pro-apoptotic effect of *TP53* through the enhancement of its acetylation and highlights that the function of miRNAs as oncogenes or tumor suppressor genes depends on both the cellular context and the class of chemotherapeutics used.

Despite various obvious caveats, several laboratories have generated some striking data. They explored how microRNAs manipulate drug resistance to cause tumor relapse/progression or their capacity to enhance sensibility to chemo-treatments [203,204,205,206,207,208]. Adding a further layer of complexity, however, single miRNAs can also act pleiotropically to target different pathways simultaneously, frequently in the context of a network. This unique ability to regulate multiple genes changes the traditional “one drug-one target” paradigm to a new “one drug-multiple targets” paradigm, and understanding of these miRNA networks will indicate how to get the best benefits for patients.

## 8. Conclusions

In principle, targeting the pathways of lymphoid malignancies as well as other cancers by miRNA-based interventions can provide a powerful therapeutic approach. However, despite the encouraging results obtained in preclinical models and the availability of the Phase I clinical trial with miR-34a mimic, there are still many issues that need to be addressed for an effective translation into clinical practice. Several substantial obstacles currently exist concerning potential off-target effects, efficacy and safety, which slow down progress toward clinical application. One of the main problems to be solved depends on the pleiotropic and sometimes generic activity of miRNAs; it is very difficult to identify targets that are specific to a particular type of cancer, also in consideration of the tissue specificity and kinetics of miRNA expression. Indeed, the expression of miRome changes dynamically during cell functions, and the same miRNA can act on several cell lineages exerting absolutely different functions. Furthermore it can operate as either a tumor suppressor or an oncogene depending on the cellular context and the expression of the target genes within that cell. For example, miR-222 acts as an oncomir overexpressed in gastric-carcinoma where it targets the tumor suppressor *PTEN* [209]. Conversely, it is down-regulated in erythroblast leukaemia since it operates as a tumor suppressor gene targeting the oncogenic c-KIT protein during erythroid differentiation and maturation as consequence its persistence down-regulation probably unblocks KIT expression causing the expansion of erythroblasts [210]. Therefore, it is very difficult to properly modulate the expression of a specific miRNA in malignant cells without the risk of unwanted and potentially harmful effects in another cellular context, and it is crucial to deliver miRNA-based drugs (either antagomirs or mimic miRNAs) only into the intended target tissues. This means that relevant hurdles to solve are how to release these drugs into specific tissues, and consequently how to establish their appropriate dosage, with a dynamic adjustment depending on their pharmacodynamic parameters.

Because lymphoid malignancies are classified according to the origin of the neoplastic cells, resulting “frozen” at a particular differentiation stage that reflects their origin through a distinct and specific miRNA profile, the assessment of miRNA expression may be a powerful diagnostic tool. In particular, some miRNAs are specific markers of lymphoma type and their use is especially advantageous in borderline cases in which different miRNA profile could help to distinguish between two diverse entities. In addition, some miRNAs are markers of disease response and are therefore useful to evaluate the effectiveness of therapy and to potentially assist clinical decision making as unique accessible biomarkers to detect and monitor lymphomas.

In conclusion, the last few years have seen an explosive growth of knowledge and a remarkable progress in the understanding of the molecular mechanisms and networks regulated by miRNAs. These molecules are increasingly attractive to clinical practice and the intriguing and promising perspectives of their use in oncology are expected to become ever more realistic and accessible.
